# Transcranial Random Noise Stimulation Modulates Neural Processing of Sensory and Motor Circuits, from Potential Cellular Mechanisms to Behavior: A Scoping Review

**DOI:** 10.1523/ENEURO.0248-21.2021

**Published:** 2022-01-06

**Authors:** Weronika Potok, Onno van der Groen, Marc Bächinger, Dylan Edwards, Nicole Wenderoth

**Affiliations:** 1 Neural Control of Movement Lab, Department of Health Sciences and Technology, ETH Zurich, 8093, Zurich, Switzerland; 2 Neuroscience Center Zurich (ZNZ), University of Zurich, Federal Institute of Technology Zurich, University and Balgrist Hospital Zurich, Zurich 8057, Switzerland; 3 Future Health Technologies, Singapore-ETH Centre, Campus for Research Excellence and Technological Enterprise (CREATE), 138602, Singapore; 4 Neurorehabilitation and Robotics Laboratory, School of Medical and Health Sciences, Edith Cowan University, Joondalup, Western Australia 6027, Australia; 5 Moss Rehabilitation Research Institute, Elkins Park, PA 19027

**Keywords:** neuromodulation, noise benefits, sensory and motor systems, stochastic resonance, transcranial random noise stimulation, voltage-gated sodium ion channels

## Abstract

Noise introduced in the human nervous system from cellular to systems levels can have a major impact on signal processing. Using transcranial stimulation, electrical noise can be added to cortical circuits to modulate neuronal activity and enhance function in the healthy brain and in neurologic patients. Transcranial random noise stimulation (tRNS) is a promising technique that is less well understood than other non-invasive neuromodulatory methods. The aim of the present scoping review is to collate published evidence on the effects of electrical noise at the cellular, systems, and behavioral levels, and discuss how this emerging method might be harnessed to augment perceptual and motor functioning of the human nervous system. Online databases were used to identify papers published in 2008–2021 using tRNS in humans, from which we identified 70 publications focusing on sensory and motor function. Additionally, we interpret the existing evidence by referring to articles investigating the effects of noise stimulation in animal and subcellular models. We review physiological and behavioral findings of tRNS-induced offline after-effects and acute online benefits which manifest immediately when tRNS is applied to sensory or motor cortices. We link these results to evidence showing that activity of voltage-gated sodium ion channels might be an important cellular substrate for mediating these tRNS effects. We argue that tRNS might make neural signal transmission and processing within neuronal populations more efficient, which could contribute to both (1) offline after-effects in the form of a prolonged increase in cortical excitability and (2) acute online noise benefits when computations rely on weak inputs.

## Significance Statement

Transcranial random noise stimulation (tRNS) is an emerging non-invasive stimulation method that adds electrical noise to cortical circuits to modulate physiology and behavior. Our analysis reveals that tRNS can enhance neural processing which manifests either as (1) offline after-effects following prolonged stimulation or (2) acute online noise benefits immediately during stimulation. We synthesize evidence derived from behavioral, physiological and single cell studies, and argue that tRNS is unlikely to act on synaptic plasticity per se but rather modulates neuronal excitability via voltage-gated sodium channels. We further propose that acute online noise benefits result from increasing the signal-to-noise ratio of the stimulated area, particularly, in response to weak inputs.

## Introduction

Transcranial random noise stimulation (tRNS) is a transcranial electrical stimulation (tES) modality which has received increasing scientific attention during the last decade (Paulus, 2011; Miniussi et al., 2013; Antal and Herrmann, 2016; Antal et al., 2016; Fertonani and Miniussi, 2017). Here, we review the available evidence on how tRNS might modulate neural processing within cortical sensory or motor systems. The majority of previous studies using tRNS, stimulated the brain continuously for several minutes, investigating both physiological and behavioral after-effects. More recently acute effects of tRNS have also been explored, showing that tRNS can exert immediate neuromodulatory effects. Although it is not completely clear which biological substrate underpins these effects, experiments using pharmacological interventions or specific preparations in animals have generated testable hypotheses of how tRNS modulates neuronal function.

In this scoping review we focus on the effects of tRNS on sensory and motor functions. We first provide a summary of tRNS properties. We then summarize evidence showing that tRNS modulates physiological and behavioral outcome parameters either in from of offline after-effects, i.e., changes which are measured after prolonged continuous tRNS application, or in the form of acute online effects which are immediately observable when tRNS is applied.

## Materials and Methods

**Figure 1.**
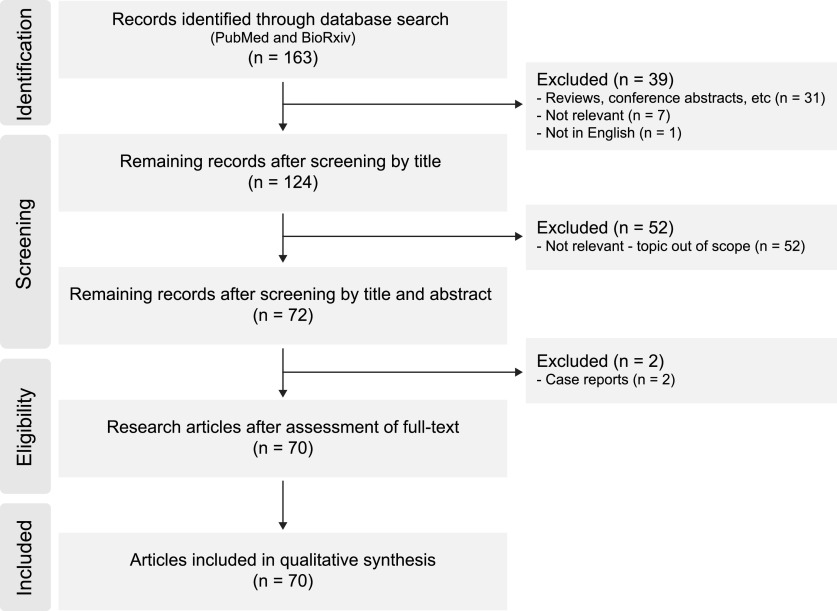
Data charting process.

We followed a reviewing process according to the PRISMA guidelines extension for scoping reviews (Tricco et al., 2018). Our central goal was to synthesize the effects of tRNS on sensory and motor function in humans. To address this, we defined our eligibility criteria as primary studies published after 2008 (the year when tRNS was first introduced), written in English, investigating modulation of sensory or motor function using tRNS in humans. Our search was conducted using the PubMed and BioRxiv databases, with the search phrase “transcranial random noise stimulation.” From this search we only included research articles describing studies using tRNS to modulate sensory and motor functions in humans. We screened the identified articles first based on the titles, then abstracts and finally the full-text. From the initial search pool (163 titles found), we excluded non-research articles (reviews and conference abstracts), studies that did not use tRNS or were not written in English. We then screened the remaining tRNS research articles to exclude all studies that did not concern sensory or motor functions. In the last screening step, we removed case reports. The search process is summarized in Figure 1.

Screening identified 163 papers of which 70 met the criteria. In order to interpret these studies and integrate the provided insights into the broader concept of non-invasive brain stimulation, we refer to additional literature that (1) has investigated the effect of electrical noise in animal models or (2) has used other forms of electrical brain stimulation. This review protocol was not preregistered.

## Results

We reviewed 70 articles investigating tRNS effects on sensory and motor function in human participants. We divide the eligible studies into those measured with physiology or behavior, assessing either offline after-effects and learning or acute online effects.

We found 19 articles focusing on the physiological effects of tRNS in healthy individuals. 18 studies investigated offline after-effects in excitability of the primary motor cortex (M1; *N* = 15), primary visual cortex (V1; *N* = 1), and auditory cortex (AC; *N* = 2). One study tested the acute online effects of tRNS on M1 excitability.

A total of 50 reviewed studies investigated whether tRNS modulates behavior. Of these studies, 31 in the visual (*N* = 7), somatosensory (*N* = 1), and motor (*N* = 6) systems examined offline after-effects or learning following tRNS in healthy volunteers. Moreover, offline after-effects on visual (*N* = 5), auditory (*N* = 7), and pain and motor function (*N* = 5) were tested in clinical populations. Twenty studies focused on the acute online effects of tRNS on visual (*N* = 10) and auditory processing (*N* = 5), somatosensation caused by stimulation (*N* = 2), and motor function (*N* = 1) in healthy volunteers. Again, acute online effects on visual (*N* = 1) and motor function (*N* = 1) were also tested in clinical populations.

The purpose, methodology and main findings of each tRNS study in humans are summarized in the Table provided as Extended Data 1. We further combine the evidence from the reviewed studies and discuss the findings in the context of potential underlying mechanisms in Discussion.

10.1523/ENEURO.0248-21.2021.ed1 Extended Data 1 Summary of the purpose, stimulation parameters and findings of tRNS and RNS studies. Download Extended Data 1, DOC file.

## Discussion

This scoping review found 70 primary research studies that investigated the effects of tRNS on sensory and motor function in humans. Here, we first discuss a summary of tRNS properties. We then synthesize the findings from studies examining tRNS modulation of physiological and behavioral outcome parameters measured either as offline after-effects and learning effects, or acute online effects during tRNS. We interpret this evidence by referring to additional literature on the effect of electrical noise in animal models or other forms of ES.

### Stimulation properties

**Figure 2.**
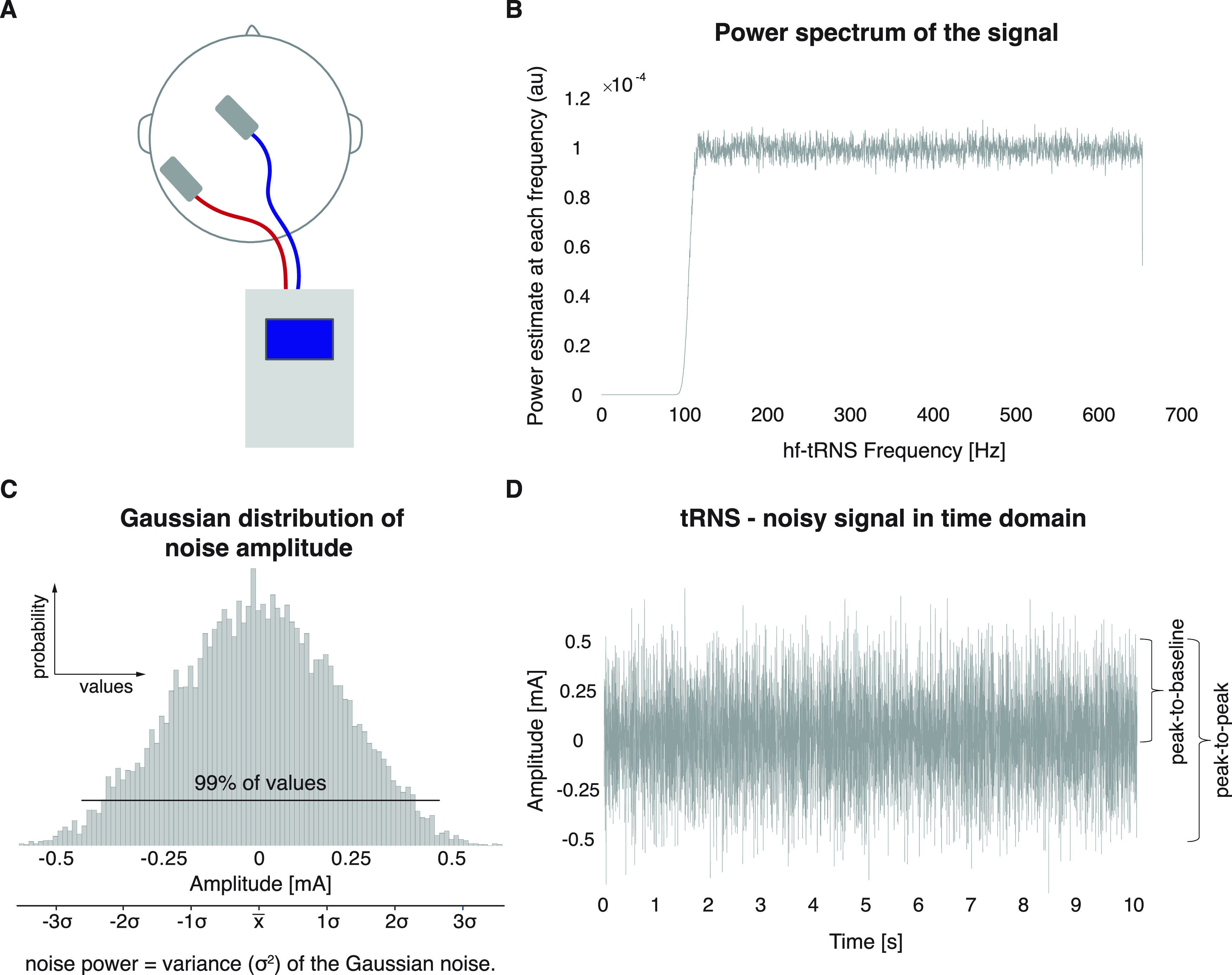
***A***, Example of a tRNS montage. The battery-driven stimulator applies current which travels in a biphasic manner between two stimulation electrodes (positioned anterior and posterior to the M1; Rawji et al., 2018; Potok et al., 2021), resulting in polarity independent stimulation (Pirulli et al., 2016). ***B***, Power spectrum of a typical tRNS signal, shown for hf-tRNS (101–640 Hz). The signal can be characterized as “white noise”, i.e., power is approximately constant for all frequencies. ***C***, The random current intensities are normally distributed with 99% of the values lying between the peak-to-peak amplitude (see ***D***). The noise power can be expressed as the variance of the signal. ***D***, tRNS signal in the time domain. Stimulation intensity is traditionally described as the peak-to-baseline or peak-to-peak amplitude of the current output signal. This example shows a tRNS signal with the frequently used intensity of 1-mA peak-to-peak (Terney et al., 2008; Parkin et al., 2019; Qi et al., 2019).

During tRNS, alternating currents travel between two electrodes with constantly changing polarity (Pirulli et al., 2016; Fig. 2*A*). The biphasic sinusoidal current is delivered at random frequencies within a predefined range and can be described as “white noise”, i.e., the induced power spectral density (the squared amplitude for a given frequency band) is constant for all frequencies (Fig. 2*B*). The maximum frequency range is often determined by the device and typically ranges between 0.1 and 700 Hz (Terney et al., 2008; Moret et al., 2019). Two commonly used subtypes are high-frequency tRNS (hf-tRNS; >100 Hz) and low-frequency tRNS (lf-tRNS; <100 Hz). The amplitude of tRNS signals is usually drawn from a Gaussian-distribution with a mean current of zero (Fig. 2*C*). Thus, the net effect of tRNS is 0 mA unless an offset is introduced by adding a direct current component. tRNS intensity has traditionally been reported as “peak-to-baseline” or “peak-to-peak” amplitudes (Fig. 2*D*). To allow replication and comparison across studies, it is important to explicitly state which convention is used. Additionally, it might be more informative to report the overall power of the current signal (which corresponds to the variance of the intensities distribution) rather than the maximum amplitude because of the distributed characteristic of the waveform (see Fig. 2*C*). tRNS is a safe method if used according to general safety guidelines for tES (Fertonani et al., 2015; Bikson et al., 2016, 2018; Woods et al., 2016). It has been shown that after delivering tRNS with an intensity of 1-mA peak-to-peak amplitude for 10 min, the concentration of serum neuron-specific enolase, a sensitive marker of neuronal damage, remains unchanged (Terney et al., 2008). Also, the induced discomfort because of cutaneous sensation is low in comparison to transcranial direct current stimulation (tDCS; Ambrus et al., 2010, 2011), which is advantageous for experimental blinding.

Low-intensity tES as used in human volunteers, including tRNS, is unlikely to directly elicit changes in neural spiking activity (Liu et al., 2018) since invasive recordings and modeling demonstrated that electrical fields induced by common tES protocols do not exceed 1 V/m in the brain (Opitz et al., 2015, 2016; Huang et al., 2017). However, networks of many synaptically connected active neurons reveal higher sensitivity to field modulation than a single-neuron threshold, thus, amplifying the stimulation effect (Fröhlich and McCormick, 2010; Reato et al., 2010). Therefore, even subthreshold stimulation at intensities well below the action potential threshold can substantially modulate neural activity (Gluckman et al., 1996; Francis et al., 2003; Bikson et al., 2006). It is known that the electric field induced in the brain is independent of the stimulation frequency (Vöröslakos et al., 2018). However, high frequencies might be filtered out by the neural structures (Deans et al., 2007; Liu et al., 2018) which gives rise to the concern that hf-tRNS might result in seemingly little modulation of neuronal activity. Despite the evidence showing effective modulation of single cells responses in animal models with electrical random noise (Onorato et al., 2016; Remedios et al., 2019; see below, tRNS causes acute physiological effects), the exact mechanism by which high-frequency stimulation affects neural structures is currently unknown. Nevertheless, there is a growing body of evidence in the literature for invasive ES, that high and ultra-high frequencies (>∼1 kHz) can effectively modulate neuronal activity and cause clinically meaningful effects in humans (Kilgore and Bhadra, 2014; Hottinger et al., 2016; Kapural et al., 2016; Harmsen et al., 2019), supporting the merit of transcranial application.

### tRNS causes physiological after-effects leading to increased cortical excitability

18 studies investigated the physiological after-effects of tRNS on cortical excitability. A vast majority of studies in humans have investigated whether tRNS modulates cortical excitability, as measured via motor-evoked potentials (MEPs; *N* = 15) or phosphene thresholds (*N* = 1) which were elicited by single-pulse transcranial magnetic stimulation (TMS) over motor cortex and visual cortex, respectively. Most of these studies have tested tRNS-induced after-effects, i.e., excitability was measured at baseline as well as after applying tRNS for a stimulation period of several minutes over M1. Specifically, 10 min of tRNS has been shown to increase corticospinal excitability (CSE) of M1 for up to 60 min, in both upper (Terney et al., 2008; Moliadze et al., 2012; Abe et al., 2019), lower extremities (Laczó et al., 2014), and pharyngeal muscle (Zhang et al., 2021), with occasional reports suggesting inhibitory effects for low intensities (Moliadze et al., 2012). A similar increase in excitability was observed in visual cortex where hf-tRNS decreased the TMS-evoked phosphene threshold for up to 60 min after stimulation (Herpich et al., 2018). While 5 min of tRNS is most likely the minimum stimulation duration for enhancing CSE of the motor system (Chaieb et al., 2011) it is not clear whether there is also a maximum duration which should not be exceed. Previous work suggests that hf-tRNS stimulation periods between 10 and 20 min seem to be appropriate to increase cortical excitability (Van Doren et al., 2014; Herpich et al., 2018; Parkin et al., 2019).

After-effects of tRNS have been suggested to depend on the stimulation frequency spectrum, with hf-tRNS inducing stronger after-effects than lf-tRNS (Terney et al., 2008), especially when the full hf-tRNS spectrum (100–700 Hz) is delivered (Moret et al., 2019). When directly compared with other brain stimulation methods, tRNS resulted in stronger increase in CSE than anodal tDCS (a-tDCS; Moliadze et al., 2014; Inukai et al., 2016), intermittent theta-burst stimulation (Moliadze et al., 2014), or 140-Hz transcranial alternating current stimulation (tACS; Inukai et al., 2016). It has been hypothesized that hf-tRNS including a direct current offset results in a stronger increase in CSE than hf-tRNS alone (Ho et al., 2015); however, this was only observed at a trend level and the direct comparison between stimulation conditions did not reveal significant differences.

Previous studies have used different electrode montages. When stimulating motor cortex, most studies placed one electrode over M1 and the other over the contralateral (supra)orbital cortex to modulate CSE (Terney et al., 2008; Moliadze et al., 2012, 2014; Chaieb et al., 2015; Ho et al., 2015; Inukai et al., 2016; Moret et al., 2019). This choice seems to be justified since a recent study found that applying hf-tRNS via the conventional M1/contralateral orbit montage caused larger CSE after-effects than a bilateral M1-M1 montage (i.e., targeting motor cortex of both hemispheres; Parkin et al., 2019). However, a bilateral montage might have its merit, particularly when sensory areas are stimulated. This was demonstrated for the auditory (Van Doren et al., 2014) and visual domain (Herpich et al., 2018) where delivering hf-tRNS bilaterally, i.e., with the electrodes placed on both hemispheres, was shown to enhance cortical excitability. The effectiveness of a bilateral montage was further demonstrated by several studies which tested the effect of tRNS on sensory detection tasks (see below, tRNS induces behavioral after-effects and modulates perceptual learning and motor function in health and disease and also see below, tRNS acutely affects perceptual and motor performance). A recent study (Potok et al., 2021) used an unilateral electrode montage positioned anterior and posterior to the M1 (45° away from the nasion-inion mid-sagittal line; Rawji et al., 2018). In this arrangement current oscillates perpendicular to the central sulcus, which has been hypothesized to be more efficient in modulating cortico-spinal excitability (Rawji et al., 2018). Additionally, this montage enables positioning the TMS coil directly on the scalp and not on top of the electrode. One study showed that the effects of tRNS on the targeted area seem to be dependent on the distance between the electrodes (Moliadze et al., 2010). To obtain the optimal electrode placement for targeted brain stimulation, it is highly recommended to use electric field modeling (Bikson et al., 2018; Bergmann and Hartwigsen, 2021). Note, however, that until now there is no software providing a reliable simulation of the electric field induced when current waveform of variable intensities and frequencies are used, as with tRNS.

Physiologic after-effects outside of motor or visual cortex are less well understood. Studies investigating cortical excitability within AC (*N* = 2) show contradictory evidence regarding tRNS influence on auditory steady state responses measured with EEG (Van Doren et al., 2014; Schoisswohl et al., 2021).

Similar to other tES paradigms, there is a variability in effectiveness across tRNS studies and study populations. It is currently unclear whether these variable result patterns reflect small or inconsistent effects induced by tRNS or depend on participant-specific determinants. For example, the after-effects following tRNS were suggested to vary depending on interindividual differences such as age (Fertonani et al., 2019) or a person’s susceptibility to placebo effects (Kortuem et al., 2019), but probably independent of the BDNF gene polymorphism (Antal et al., 2010). Long-term modulation of CSE with tRNS was suggested to be task-dependent and specific to the underlying brain state (Chaieb et al., 2009; Saiote et al., 2013; Jooss et al., 2019; Qi et al., 2019). However, many of these potential participant-specific determinants still await replication.

It is not fully understood which biological mechanism underlies long-lasting physiological effects of tRNS. A first pharmacological pilot study suggests that the facilitatory effects of tRNS are supressed by a voltage-gated sodium channel blocker, as well as by a GABA_A_ receptor agonist, while they were unaffected by NMDA receptor antagonists (Chaieb et al., 2015). In line with the proposal that tRNS modulates excitatory circuits it has been shown that tRNS increases intracortical facilitation in motor cortex (Terney et al., 2008) and evoked responses in somatosensory cortex (Saito et al., 2019). Evidence for the potential involvement of a GABAergic mechanism is, however, much more mixed. A recent animal study investigated histologic changes after chronic tRNS in juvenile mice (Sánchez-León et al., 2021). After nine tRNS sessions, each lasting 20 min, GABA levels (quantified via GAD65-67 immunoreactivity markers) were decreased suggesting that cortical disinhibition might contribute to tRNS-induced effects. However, studies investigated the activity of GABAergic inhibitory circuits after a single session of tRNS did not support the hypothesis that a reduction of GABA_A_ and GABA_B_ mediated inhibition (Ho et al., 2015; Terney et al., 2008; Saito et al., 2019) contributes to after-effects on excitability in M1.

Taken together, tRNS-induced after-effects seem to rely on a mechanism which is probably not driven by NMDA-receptor activity. High GABA_A_ activity has been shown to prevent tRNS effects to be expressed, however, evidence is mixed as to whether tRNS modulates cortical excitability via a GABAergic disinhibition mechanism. The strongest evidence up-to-date is that tRNS-induced after-effects are associated with increased activity within facilitatory cortical circuits which might facilitate neural transmission at the population level, thereby bringing the cortex into a plasticity-supporting state. At the cellular level this might be achieved by modulating the transmission at voltage-gated sodium channels, however, most of the available evidence supporting this mechanism was obtained when RNS was acutely applied as discussed next.

### tRNS causes acute physiological effects

The majority of the available neurophysiological studies in humans investigated tRNS-induced after-effects on cortical excitability, while only one study tested the acute physiological effects of tRNS (Potok et al., 2021). By contrast, many *in vitro* studies and research in animal models have focused on how neural activity is changed during RNS. For example, it has been demonstrated that electrical RNS increases action potential firing in mouse primary sensory neurons of dorsal root ganglia in response to weak stimuli (Onorato et al., 2016). One likely cellular substrate for mediating this acute RNS effect are voltage-dependent ion channels. Externally applied electrical white noise was shown to increase the signal transduction capacity at a subcellular level in artificial lipid bilayers, where it facilitated openings of voltage-dependent alamethicin ion channels (Bezrukov and Vodyanoy, 1995, 1997). Additionally, it has been demonstrated that subthreshold ES opens sodium channels, causing a small influx of Na^+^, which in turn causes a rapid, local depolarization of the cell membrane. Repolarization, by contrast, is a passive process which occurs over a longer time period. If the ES is quickly repeated, as may be the case with tRNS, multiple Na^+^ influxes occur in rapid succession and the membrane potential is gradually shifted toward depolarization (Schoen and Fromherz, 2008). An alternative account for how RNS affects sodium channels was provided by a recent study which directly probed whether applying RNS simultaneously with voltage-clamp ramps, affects the kinetics and peak amplitude of Na^+^ currents in rat somatosensory and auditory pyramidal neurons *in vitro* (Remedios et al., 2019). One main finding of this study was that the observed RNS effects can be explained by modulating the kinetics of activation and inactivation of Na^+^ channels as demonstrated by a Hodgkin–Huxley neuron model which replicated the experimental data.

**Figure 3.**
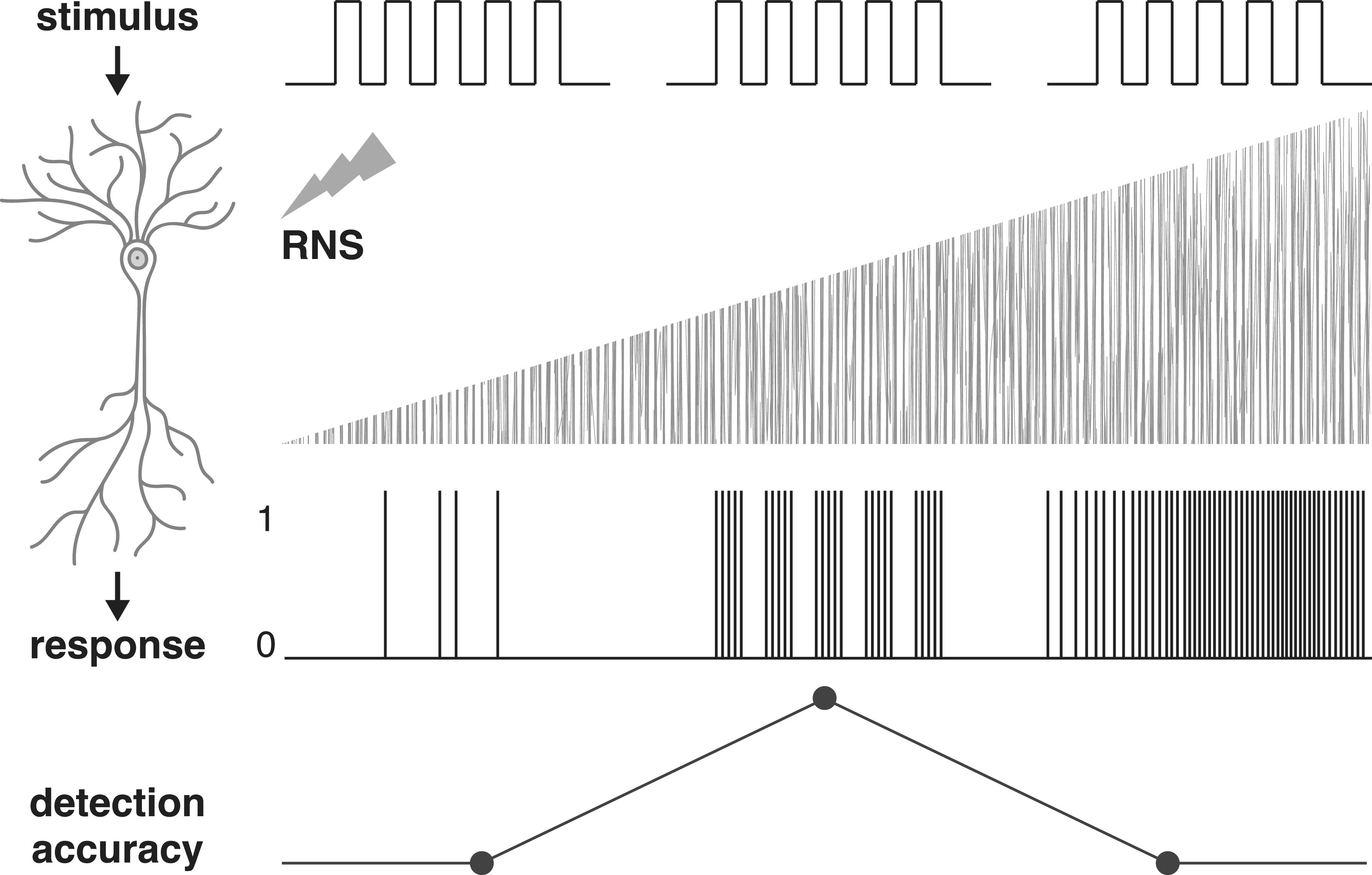
Conceptual representation of how electrical RNS may enhance the neural signal and influence neural response according to the SR phenomenon. Weak stimuli of depolarizing steps are delivered to a cell accompanied by white electrical RNS of increasing power (low, optimal, or excessive noise level). Stimuli evoke passive changes in membrane potentials resulting in a binary output response when the membrane potential reaches a response threshold. Stimulus input combined with a low level of noise is too weak to evoke an accurate response. For stimuli accompanied by an optimal level of noise, the output response corresponds to the exact timing of input stimuli. Excessive noise added to the stimuli results in false alarms in the output response. Detection accuracy of cell firing according to the stimulus is enhanced during the optimal level of noise delivery.

Most of the above studies were motivated by the idea that neurons might be sensitive to the stochastic resonance (SR) phenomenon (McDonnell and Abbott, 2009; see Box 1). SR describes that the response of nonlinear systems to weak, subthreshold signals can be enhanced by adding an optimal level of random noise (McDonnell and Abbott, 2009; Fig. 3). The SR mechanism has been confirmed for neural systems and has been argued to be beneficial for neural processing by increasing the signal-to-noise ratio. For example, potentials evoked by experimental stimuli were enhanced by the optimal level of optogenetic noise photostimulation (Huidobro et al., 2017), acting on the Na^+^ current (Mabil et al., 2020). Furthermore, the electrical RNS delivered to neurons in rat hippocampal slices increased extracellular electrical activity (Gluckman et al., 1996) and enhanced their firing activity for a particular noise level (Stacey and Durand, 2000). Similarly, subthreshold sinusoidal and stochastic noise can modulate the sensitivity of individual neurons in the medial vestibular nucleus without affecting basal firing rates (Stefani et al., 2019). Finally the two studies cited above (Onorato et al., 2016; Remedios et al., 2019) further demonstrated that neural responses to externally applied stimuli were maximally enhanced when an optimal level of electrical RNS was applied and linked this effect specifically to the induced Na^+^ current.

Box 1 Definitions of SR phenomenon and nonlinear system.

Stochastic resonance (SR) describes any phenomenon where the presence of noise in a nonlinear system is better for output signal quality than its absence (McDonnell and Abbott, 2009). In a nonlinear system the change of the output is not proportional to the change of the input. A good example of a nonlinear system is a neuron, where any stimulus or input signal needs to reach certain threshold in order to evoke an action potential response. One key indicator of the SR phenomenon in a broad sense is that the investigated system “benefits” from noise, which usually refers to better detection, transmission or processing of the input signal than when no noise is present. In its simplest manifestation, SR results from the concurrence of a threshold, a subthreshold stimulus, and noise (Gingl et al., 1995). Another SR feature is that noise benefits are a function of noise intensity exhibiting an inverted U-shape dose-response relationship. It refers to the assumption that there is an optimal noise level for enhancing the response of nonlinear systems to weak subthreshold signals, where too low noise does not change the system output and excessive noise degrades performance of the system (e.g., by causing false alarms). It was recently suggested that tRNS can be used as a tool to investigate the SR principle in the human cortex (van der Groen and Wenderoth, 2016).

In humans, only one study demonstrated acute online physiological effects of hf-tRNS. It manifested as an immediate decrease in the resting motor threshold measured with TMS, reflecting the modulation of responsiveness of M1 during very brief hf-tRNS delivery (Potok et al., 2021). This study demonstrated that tRNS can acutely generate noise benefits by enhancing the response of neural populations in human M1 for near-threshold TMS, in line with predictions of SR. Interestingly, pharmacological studies have suggested that activity of the voltage-gated sodium channels is an important determinant of motor threshold (Tergau et al., 2003; Sommer et al., 2012; Ziemann et al., 2015). These findings further support the hypothesis that high-frequency electrical RNS modulates sodium currents.

Taken together, tRNS might acutely modulate voltage-gated sodium channels. This might (1) cause multiple small Na^+^ influxes which are accumulated such that the membrane potential is biased toward depolarization or (2) change the kinetics of Na^+^ channel activation/inactivation.

In accordance with the SR mechanism, applying an optimal level of electrical noise generates immediate noise benefits such that the cell becomes more responsive to weaker external stimuli than when no noise is added. It has been suggested that these effects might propagate from the single cell to the neuronal population level such that RNS causes large cell ensembles to synchronize their firing (Fröhlich and McCormick, 2010; Reato et al., 2010), thereby increasing the signal-to-noise ratio (Miniussi et al., 2013) and/or enhancing cortical responsiveness (Potok et al., 2021).

### tRNS induces behavioral after-effects and modulates perceptual learning and motor function in health and disease

Several studies (*N* = 11) have investigated whether tRNS modulates perceptual learning when applied during the training period. A seminal study showed that 22 min of hf-tRNS applied to V1 during a visual perceptual learning task improved orientation discrimination accuracy significantly more than lf-tRNS, a-tDCS, cathodal tDCS, sham, or an active control condition where tRNS was applied to the vertex (Fertonani et al., 2011). Other study replicated and extended these findings by showing that hf-tRNS facilitates perceptual learning only when applied during the learning period and, unlike a-tDCS, not when applied solely beforehand (Pirulli et al., 2013), indicating that mechanisms of action might differ between tRNS and a-tDCS. Similar benefits were demonstrated for other visual learning tasks such that applying hf-tRNS over V1 during training decreased the peripheral crowding threshold (Contemori et al., 2019) and led to fast improvements in a motion discrimination task (Herpich et al., 2019). Likewise, tRNS effects were also reported for visual training paradigms in neurologic patients. A series of experiments investigated boosting effects of visual training coupled with hf-tRNS of V1 on visual perceptual learning in individuals with mild myopia (Camilleri et al., 2014, 2016), amblyopia (Campana et al., 2014; Moret et al., 2018; Donkor et al., 2021) and chronic cortical blindness (Herpich et al., 2019). The recovery of contrast sensitivity, visual acuity, and motion processing observed in these experiments suggested the potential of combining visual training with tRNS to help restoring damaged visual abilities for divergent visual dysfunctions. These positive effects seemed to result from enhanced training efficacy because of tRNS (Camilleri et al., 2014, 2016; Moret et al., 2018; Herpich et al., 2019; Donkor et al., 2021). For example, in patients with mild myopia the effects of two weeks of visual training combined with tRNS were comparable to eight weeks of solely training (Camilleri et al., 2014) and the improvement in cortical blindness patients after 10 d, was comparable to around two months of training only (Herpich et al., 2019).

While the above studies applied tRNS in combination with a perceptual task, others applied tRNS during rest and investigated whether behavioral after-effects were induced (*N* = 2). Offline hf-tRNS applied over parieto-occipital cortex was shown to induce moderate aftereffects in γ-range brain oscillatory activity measured with EEG during motion direction discrimination task performance. These physiological effects were, however, not accompanied by behavioral task performance modulation (Ghin et al., 2021). Saito et al. (2019) showed that tRNS applied without training, improved tactile spatial discrimination task performance illustrated by a decreased threshold in discriminating grating orientation after 10 min of stimulation. The stimulation affected early processing in the primary somatosensory cortex, modulating neuronal activity by increasing the N20 sensory-evoked potential amplitude, that indicates an increase in cortical excitability (Saito et al., 2019).

In the auditory domain, tRNS effects were mainly tested in patients (*N* = 7). Lf-tRNS was demonstrated to induce a large transient suppressive effect on tinnitus loudness and tinnitus-related distress (Vanneste et al., 2013; Joos et al., 2015), outperforming tDCS and α tACS (Vanneste et al., 2013). Moreover, studies investigating lf-tRNS delivered over dorsolateral prefrontal cortex and AC showed the superiority of multisite treatment protocols (To et al., 2017; Mohsen et al., 2018), and multiple sessions (Mohsen et al., 2019b) over sham, one-site or single-session interventions. Lf-tRNS after-effects were illustrated by increased α activity that serves an inhibiting role and is usually decreased in AC of tinnitus patients. Such increase in inhibiting α activity most probably leads to a reduction in the hyperexcitability of the AC and thus, a decrease in tinnitus symptoms (Mohsen et al., 2019a). Despite increasing evidence for the efficacy of lf-tRNS in reducing tinnitus symptoms, the differences in treatment responders suggested the need for individualized treatment procedures, especially when hf-tRNS is used (Kreuzer et al., 2019).

Furthermore, six studies showed that tRNS also has potential to influence motor performance. tRNS applied at rest improved performance in a visuomotor tracking task (Abe et al., 2019). When delivered during several blocks of a serial reaction time task, tRNS shortened the response times (Terney et al., 2008). Interestingly, lf-tRNS and hf-tRNS were shown to modulate visuomotor learning differentially with hf-tRNS improving and lf-tRNS hindering performance (Saiote et al., 2013). Further, an improvement of complex continuous tracing task performance with the non-dominant hand was observed during both hf-tRNS and a-tDCS (Prichard et al., 2014). The time course of skill gains differed between stimulation types, suggesting likely different mechanisms by which each distinct tES protocol influences motor learning. However, application of hf-tRNS failed to enhance skill acquisition and retention in a golf putting task (De Albuquerque et al., 2019). In this regard, a recent study investigating the effects of motor training in combination with tRNS provided at various timepoints (before, during, or after training vs sham) failed to observe differences between these conditions on motor learning (Hoshi et al., 2021). There is also preliminary evidence (*N* = 5) indicating a potentially beneficial influence of tRNS on motor control, pain or perceived motor fatigue in Parkinson’s disease (Stephani et al., 2011; Monastero et al., 2020), relapsing-remitting multiple sclerosis (Palm et al., 2016; Salemi et al., 2019) and subacute ischemic stroke patients (Arnao et al., 2019). However, further research including studies of greater sample size is required to confirm the observed effects and fully understand their underlying mechanisms.

To this end, the exact mechanism by which tRNS induces long-term behavioral after-effects is not clear. So far, only one study directly linked behavioral after-effects with larger excitability showing increased sensory discrimination performance and greater SEP amplitude after tRNS (Saito et al., 2019). For studies where tRNS was applied together with a learning task, it is difficult to disentangle whether the long-term performance enhancement is caused by tRNS acting on synaptic neuroplasticity per se, or rather on preventing homeostasis of the system or increasing the signal-to-noise ratio for task-related neural activity (Fertonani et al., 2011). Moreover, although the investigated tRNS-induced modulation seems to be consistent across healthy individuals and patients, one needs to keep in mind that in neurologic diseases transmitter availability as well as other functional and structural brain features might differ on a qualitative level and have an impact on the efficacy of non-invasive brain stimulation to alter brain function.

### tRNS acutely affects perceptual and motor performance

A series of recent studies focusing on the immediate, i.e., online effects of tRNS on behavior investigated whether perceptual and motor tasks can be acutely improved by hf-tRNS (*N* = 20).

For visual tasks (*N* = 10), hf-tRNS acutely increases sensitivity for low contrast visual stimuli as demonstrated for contrast detection task (van der Groen and Wenderoth, 2016), orientation discrimination task (Melnick et al., 2020), lateral visual masking protocols (Battaglini et al., 2019), and exploring stimulation effects using visual stimuli with various properties (Battaglini et al., 2020). It was further shown that delivering central noise via hf-tRNS influences state-switching dynamics of binocular rivalry (van der Groen et al., 2019) and accelerates perceptual decision-making in a motion discrimination task (Campana et al., 2016; Ghin et al., 2018; van der Groen et al., 2018; Pavan et al., 2019; O’Hare et al., 2021).

Hf-tRNS was also shown to increase auditory detection (*N* = 4), potentially by influencing early sensory processing as indicted by reducing peak latencies of auditory event-related potentials (Rufener et al., 2017, 2018). There is evidence indicating that hf-tRNS can modulate auditory perception more efficiently than tDCS (Prete et al., 2017) and with higher effectiveness when a bilateral rather than an unilateral montage is used (Prete et al., 2018). These results need to be treated with caution, however, as a recent study questioned the beneficial effects of noise in human auditory perception. The authors did not observe improvements in the detection of acoustic stimuli in the presence of noise, regardless of whether noise was provided in an acoustic or electrical (tRNS) modality (Rufener et al., 2020).

Regarding the motor domain, applying hf-tRNS during an inhibitory “go/no-go” motor task was shown to modulate task performance by a shift in the speed-accuracy trade-off, reflected in slower reaction time and increased accuracy (Jooss et al., 2019).

A first proof-of-concept study has applied tRNS to ipsilesional M1 of stroke survivors, however, clinically relevant improvements varied across individuals and appeared to be independent of stimulation (Hayward et al., 2017). Further research in patients is needed to explore whether tRNS can boost recovery but the rationale for applying tRNS should be matched to the treatment target (Hayward et al., 2017; O’Hare et al., 2021). For example, enhancing CSE during strength training targeted at reducing arm weakness, or augmenting learning consolidation during skill practice.

How can these behavioral benefits of acute tRNS be explained? Many of the above studies were motivated by the idea that the brain responds to acute electrical noise stimulation according to the SR phenomenon (see Box 1; see above, tRNS causes acute physiological effects). The SR hypothesis makes three important predictions: first, there are “noise benefits”, i.e., adding noise makes the neural system more responsive to external stimuli as indicated by higher detection rates or lower perceptual thresholds. Second, noise benefits depend on the noise intensity according to an inverted U-shaped function (Moss et al., 2004; McDonnell and Abbott, 2009), i.e., the largest noise benefit is observed for an optimal tRNS intensity while too high or too low tRNS results in smaller or no benefits. Third, noise benefits are particularly pronounced when the neural system processes near-threshold stimuli (Gingl et al., 1995; although SR effects can also occur for suprathreshold stimuli).

Indeed, the studies cited above could show some “noise benefits” such that performance improved in the presence of tRNS relative to a baseline condition where no tRNS was applied. In line with the second prediction of SR theory, several studies have shown that hf-tRNS at optimal intensity causes performance enhancement while applying higher intensities had a detrimental effect (van der Groen and Wenderoth, 2016; van der Groen et al., 2018; Pavan et al., 2019). Finally, some perceptual detection studies compared tRNS effects for subthreshold directly with suprathreshold stimuli. These studies revealed that noise stimulation was particularly beneficial for near-threshold signals (van der Groen and Wenderoth, 2016; Rufener et al., 2017; van der Groen et al., 2018; Battaglini et al., 2019), which is in line with the third prediction of SR theory.

Although tRNS has been shown to affect behavior in accordance with SR for some tasks, it is still not clear which aspect of signal processing has been modulated. A study using drift diffusion framework (DDM) revealed that hf-tRNS-induced performance improvement in perceptual decision-making was accompanied by the increased drift-rate parameter (van der Groen et al., 2018). In DDM, the drift rate reflects the rate at which sensory evidence is accumulated (Ratcliff and McKoon, 2008). Performance improvement during tRNS was, therefore, suggested to occur via an increase in the rate of evidence accumulation, reflecting an enhancement in the quality of sensory information on which the decision is based (McIntosh and Mehring, 2017; van der Groen et al., 2018). Interestingly, equivalent noise analysis, a paradigm allowing to parcel motion perception into independent estimates of local and global processing (Dakin et al., 2005) was used to determine whether hf-tRNS modulates internal noise or global sampling (Ghin et al., 2018; Pavan et al., 2019). In this paradigm, internal noise would affect the precision of estimating each moving dot’s direction (local processing), whereas sampling reflects the number of such estimates that can be averaged (global processing; Dakin et al., 2005). It revealed that hf-tRNS influences sampling, indicating mechanisms modulating effectiveness of perceptual integration of the signal (Ghin et al., 2018; Pavan et al., 2019). In either case, effectiveness of the signal perception but not change of the decision criterion was postulated to be responsible for boosting task performance.

### Conclusions and outlook

There is growing evidence coming from behavioral, physiological, and cell studies demonstrating beneficial influence of electrical RNS on sensory or motor processing manifested either as after-effects following prolonged stimulation or as acute noise benefits. tRNS after-effects manifest as increased cortical excitability and performance improvements for selected tasks, however, there is no evidence that tRNS might act on synaptic plasticity per se. Rather, it seems to act via voltage-gated sodium ion channels in large neuronal populations. This might bring the brain into a slightly facilitated state which is beneficial for neuroplastic changes to occur. The activity of voltage-gated sodium channels has also been proposed to underlie acute noise benefits which manifest as increased effectiveness of responding to weak input signals as tRNS might improve the signal-to-noise ratio of the stimulated neuronal populations.

However, more research is needed to fully understand the neurobiological underpinnings of tRNS which can then inform the design of stimulation protocols to improve sensory and motor function in health and disease. In this regard, there are still several open questions that need to be addressed. So far tRNS effects were shown for stimulation delivered over different cortical areas. However, it remains unknown whether tRNS-induced modulation depends on the neuronal population level of the stimulation delivery within a certain system (e.g., retina vs V1 in the visual system or M1 vs spinal cord in the motor system). Moreover, effectiveness of the current stimulation may vary depending on individual differences in anatomy and could be addressed by individualizing electrode montage or stimulation intensities based on the simulations of the induced electric field. According to the SR theory, the level of noise added to the system needs to be optimized for the individual and task type to improve performance (Moss et al., 2004; McDonnell and Abbott, 2009; van der Groen and Wenderoth, 2016). It is therefore important to consider both these aspects in tRNS study designs.

Although many studies have demonstrated physiological or behavioral after-effects of tRNS consistent with neuroplastic changes, they were shown to be most probably not mediated by NMDA receptor activity (Chaieb et al., 2015). Thus, it is currently not clear how tRNS might affect synaptic plasticity. This question could be addressed by combining tRNS with other brain stimulation protocols that induce neuroplastic effects measured with electrophysiology to provide a better understanding of an underlying mechanism. Finally, as tRNS is a relatively new branch of non-invasive brain stimulation research it is difficult to assess the ratio between effective interventions and null results, the latter being likely underestimated because of the publication bias. Therefore, it is important for the field to share null findings to obtain a full and unbiased picture of the effectiveness of the tRNS method.

## Synthesis

Reviewing Editor: Julie Bakker, University of Liege Decisions are customarily a result of the Reviewing Editor and the peer reviewers coming together and discussing their recommendations until a consensus is reached. When revisions are invited, a fact-based synthesis statement explaining their decision and outlining what is needed to prepare a revision will be listed below. The following reviewer(s) agreed to reveal their identity: Elias Manjarrez, Aurore Thibaut.

2 reviewers have re-reviewed the paper and both agree that the paper has been significantly improved. However, they suggest some minor modifications, please find them below:

Reviewer 1:

-Because the authors state “from potential cellular mechanisms” in the title, it would be necessary to include in Table 1 a section termed “Potential cellular mechanisms” indicating the cellular type and animal preparation for references by Onorato et al. 2016 and Remedios et al. 2019.

-Line 325 “(note, however, that Remedios et al. reported clear noise benefits only for 1/3 of the investigated pyramidal neurons” However, this statement was not correctly interpreted from the cited study and should be removed to avoid confusion.

Reviewer 2:

The authors made substantial revisions to their manuscript which really improve the quality and impact of this review. The extensive table provided in the current version of the manuscript will be extremely useful for researchers and clinicians who plan to investigate or use tRNS.

I only have a few minor comments:

P4 l66: The authors wrote “163 positions”. Do they mean “163 results” or “titles found”?

P7 table 1: Turney et al., 2008: Remove “First introduced and” to start with “Investigated tRNS xxx” as for the other studies.

P7 table 1; column “Control stimulation parameters”. I would give more details about the parameters. Especially, for the ‘sham’ stimulation. Indeed, what is the difference between a ‘sham’ and ‘30s tRNS? 30s tRNS could be used a sham. This should be clarified.

P23: Figure 2A: Could the authors provide the electrode locations? It doesn’t seem to be a conventional montage.

P28 l264: Replace ‘random noise stimulation’ by ‘RNS’ as this acronym has been used before. I would recommend to carefully check the manuscript to make sure all acronyms are described only the first time they are used. Same for the box1 (P31), SR should be used everywhere ( ... , stochastic resonance results from the concurrence of a threshold, ... ).

P38 l489-492: This theory would benefit from being more detailed. As it is, it is unclear what it adds to the previously described hypotheses and theories.

P39 l515-517: “It is also important to keep in mind while using tRNS that according to the SR theory, the level of noise added to the system needs to be optimized for both task type and individual to improve the performance”. There is a (grammatical) problem with the first part of the sentence. Could the authors rephrase it?
