# Supplementary material for: Transcranial Random Noise Stimulation Modulates Neural Processing of Sensory and Motor Circuits, from Potential Cellular Mechanisms to Behavior: A Scoping Review
Source: eNeuro. 2022 Jan 6;9(1):ENEURO.0248-21.2021. doi: 10.1523/ENEURO.0248-21.2021 (PMC8751854; doi:10.1523/ENEURO.0248-21.2021)
Supplement: Extended Data 1 — Summary of the purpose, stimulation parameters and findings of tRNS and RNS studies. Download Extended Data 1, DOC file. [file enu-eN-REV-0248-21-s02.doc]

**Table 1 Summary of the purpose, stimulation parameters and findings of tRNS and RNS studies.**

| **Authors, year** | **Research question and study protocol** | **Participants population**  **N, gender, age (M±SD)** | **Stimulation location** | **Verum stimulation parameters** | **Control stimulation parameters** | **Main finding(s)** |
| --- | --- | --- | --- | --- | --- | --- |
| **1. Physiological effects of tRNS** | | | | | | |
| **1.1 Offline after-effects following tRNS** | | | | | | |
| Terney et al., 2008 | Investigated tRNS effects on cortical excitability and motor task performance. Measured RMT, AMT, SI_1mV_, single-pulse TMS-elicited MEPs, SICI, ICF, LICI, recruitment curves, cortical silent period, serial reaction time task performance, and EEG before and after intervention. | 80 healthy volunteers; 48F and 32M; 25.74±5.13,  age range: 20–44; all right-handed | 4x4cm electrode over left M1, 6x14cm electrode over the contralateral orbit | 1mA (peak-to-peak)  tRNS (0.1-640Hz), lf-tRNS (0.1–100 Hz), and hf-tRNS (101– 640 Hz) with 0mA offset, for 10 min in several experiments | 30s tRNS | Increased cortical excitability (probed with MEP amplitude) up to 60 min after tRNS, hf-tRNS was most effective with regard to changing the level of cortical excitability. Decreased reaction time after tRNS (in blocks 5-6 out of 14). |
| Antal et al., 2010 | Investigated whether brain-derived neurotrophic factor (BDNF) gene polymorphism is important for neuromodulation induced by iTBS, tDCS, tRNS.  Measured single-pulse TMS-elicited MEPs and SI_1mV_ before and after intervention. | 64 healthy volunteers with different BDNF gene polymorphisms (retrospective analysis from different studies); 28F, 36M; age range: 19-42 years; all right-handed | tRNS: 4x4cm electrode over left M1, 6x14cm electrode over the contralateral orbit  tDCS: 5x7cm electrodes | 1mA intensity for tRNS (0.1-640Hz) with 0mA offset, and tDCS  10 min tRNS, 7-9 min a-tDCS and 13 min c-tDCS; 2-second train of iTBS (three pulses at 50 Hz repeated at 5 Hz) was repeated every 10s; | 2 groups of different genotypes | tRNS did not affect the 2 genotype groups differently.  The impact of BDNF polymorphism on neuromodulation in humans might differ according to the mechanism of plasticity induction. |
| Moliadze et al., 2010 | Investigated the importance of the distance between stimulation electrodes, in various montages, on the ability to induce sustained cortical excitability changes using tDCS and tRNS. Measured single-pulse TMS-elicited MEPs with SI_1mV_ before, during and after intervention. | 11 healthy volunteers in tRNS experiment; 26 ± 3; all right-handed | 4x4cm electrode over left M1, 6x14cm electrode over contralateral forehead vs contralateral upper arm – for tRNS experiment | 1mA tRNS (0.1-640Hz) with 0mA offset, for  10 min per electrode montage | 2 electrode montages | The effects of tRNS on the targeted area are dependent on the distance between the electrodes. Using extracephalic reference electrodes with tES techniques, the stimulation intensity has to be adapted to account for interelectrode distance. |
| Chaieb et al., 2011 | Investigated whether a shorter duration of tRNS can induce a change in cortical excitability.  Measured RMT, AMT, SI_1mV_, and single-pulse TMS-elicited MEPs before and after intervention. | 22 healthy volunteers; 4F and 18M; age range: 20–30; 15 right-handed | 4x4cm electrode over left M1, 6x14cm electrode over contralateral orbit | 1mA hf-tRNS (101-640Hz) with 0mA offset, for  4, 5 or 6 min | 30s tRNS | A minimal stimulation duration of 5 minutes is required to induce facilitatory after-effects of tRNS on cortical excitability (increased MEP amplitude). The after-effects persist for up to 10 min. |
| Moliadze et al., 2012 | Investigated the dose-response relationship between tES intensity and the induced after-effects.  Measured single-pulse TMS-elicited MEP with SI_1mV_ before and after intervention. | 25 healthy volunteers; 6F and 19M; 25.9±2.35; age range: 23–30; all right-handed | 4x4cm electrode over left M1, 6x14cm electrode over contralateral orbit | 0.2, 0.4, 0.6, 0.8, 1mA of tRNS (0.1-640Hz) with 0mA offset and 140 Hz tACS; 6 sessions of each tES applied for 10 min on separate days, with 5s fade-in/out | sham* | Different intensities of high frequency tES result in enhancement or reduction of M1 excitability. 1mA tES results in excitation, intermediate intensity ranges of 0.6 and 0.8 mA had no effect at all, 0.4 mA induced inhibition (stronger for 140 Hz tACS than that induced by tRNS). |
| Moliadze et al., 2014 | Investigated the efficacy of three stimulation methods inducing excitability enhancement in the motor cortex: a-tDCS, iTBS, and tRNS. Measured single-pulse TMS-elicited MEPs with SI_1mV_ before and after intervention. | 12 healthy volunteers; age: 25.7±4.1; range: 23–38; all right-handed | 4x4cm electrode over left M1, 6x14cm electrode over contralateral orbit | 1mA tRNS (0.1-640Hz) and a-tDCS each for 10 min  2s train of iTBS (bursts of 3 pulses at 50 Hz repeated at 5 Hz and an intensity of 80% AMT) was repeated every 10s for a total of 190s (600 pulses) for 3 min; separate sessions counterbalanced order; double blinded | 30s tDCS | Among the stimulation methods, tRNS resulted in the strongest cortical excitability increase, and a-tDCS led to significantly longest excitability enhancement compared to sham. Different time courses of the applied stimulation methods suggest different underlying mechanisms of action. |
| Laczó et al., 2014 | Investigated the efficacy of tRNS and tDCS on the leg area of motor cortex.  Measured RMT, IO curve, single-pulse TMS-elicited MEPs with SI_1mV_ before and after intervention. | 10 healthy volunteers; 5F, 5M; 27.4±3.95, age range: 22–34; 8 right-handed | 5x7cm electrodes over leg M1, and over contralateral orbit | 2mA a-tDCS, c-tDCS, (peak-to-peak) hf-tRNS (100-640Hz) with 8s fade-in/out, applied for 10 min on separate days in randomized order (5 days in between) | 30 s of 2mA a-tDCS or c-tDCS, with an 8s fade- in/out | Leg area can be reached by weak transcranial currents. Anodal tDCS induced a constant gradual increase of cortical excitability until 60 min post-stimulation, whereas the effect of tRNS was immediate with a duration of 40 min following stimulation. Cathodal tDCS induced a decrease in MEP amplitude which did not reach statistical significance. |
| Van Doren et al., 2014 | Investigated the effects of tRNS over AC on resting state and evoked activity in healthy subjects.  Measured ASSRs with EEG during rest and auditory stimulation, before and after intervention. | 14 healthy volunteers; 7F, 7M; 24.6±1.9; all right-handed | 5x7cm electrodes over T8 and over T7; both oriented in anterior-posterior direction with the inferior center on the T position | 2 mA hf-tRNS (101-640 Hz), with 0mA offset, for 20 min with a 10s fade-in/out  1 week between verum and sham | 10s fade-in/out 2mA tRNS | tRNS increased excitability of the auditory cortex, reflected by an increased ASSR to auditory stimulation and a non-significant trend toward an increase in mean theta band power. |
| Chaieb et al., 2015 | Investigated the efficacy data with regard to the possible neuronal effect of tRNS using pharmacological intervention.  Measured RMT, AMT, SI_1mV_ and TMS-elicited MEPs before and after tRNS and a pharmacological agent vs placebo.  Pilot study. | 8 healthy volunteers; 0F, 8M; 30.1±5.2; 7 right-handed | 4x4cm electrode over left M1, 6x14cm electrode over contralateral orbit; | 1mA tRNS (0.1-640Hz) for 10 min; 6 experimental sessions were separated by a 2 weeks interval; double blinded | 5 pharmacological interventions vs placebo | Mechanism of tRNS-induced neuroplastic effects is related to sodium channel activity and γ-Aminobutyric acid type A (GABAa) receptors, but, unlike tDCS, independent of N-methyl-D-aspartate (NMDA) receptors. |
| Ho et al., 2015 | Investigated the effect of tRNS with and without a DC offset on motor cortical excitability and compared results to tDCS.  Measured single-pulse TMS-elicited MEPs, SICI and ICF before and after interventions. | 15 healthy volunteers; 7F, 8M; 24±6.07; all right-handed | 5x7cm electrodes over left M1, and over right contralateral supraorbital area | 1mA tDCS, 2mA tDCS, 2mA (peak-to-peak) hf-tRNS (100-640Hz) with 0mA offset, and 2mA (peak-to-peak) hf-tRNS + 1mA DC offset, 10 min with a 30s fade-in/out, on 5 experimental sessions (randomized order) separated by 4 days | 30s tRNS with 10s fade in | Although differences between the stimulation conditions did not reach statistical significance, the findings suggest that stimulation involving DC (tDCS and hf-tRNS including DC offset) but not solely tRNS is more likely to lead to an increase in cortical excitability. |
| Inukai et al., 2016 | Investigated the efficacy of a-tDCS, tRNS and tACS methods for increasing cortical excitability using the same subject population and same current intensity.  Measured single-pulse TMS-elicited MEPs with SI_1mV_ before and after interventions. | 15 healthy volunteers; 7F, 8M; 24±6.07; all right-handed | 5x7cm electrodes over left M1, and over right contralateral orbit | 1 mA a-tDCS, tRNS (0.1-640Hz) with 0mA offset, and 140 Hz tACS for 10 min with 10s fade-in/out  4 sessions, 3 days apart | 30s tDCS | tRNS was shown to be more effective than a-tDCS and 140Hz tACS in increasing cortical excitability compared to both pre-stimulation and sham conditions.  tRNS- and tACS-induced effects were correlated across participants. |
| Herpich et al., 2018 | Investigated whether priming the visual cortex with tRNS leads to increased and sustained excitability. Measured visual phosphenes threshold before and after intervention. | Exp.1a: 18 healthy volunteers; 11F, 7M; mean age: 22.9; all right-handed; 6 excluded  Exp.1b: 11 healthy volunteers; 7F, 4M; mean age = 20.1; all right-handed; 3 excluded  Exp.2: 22 healthy volunteers; 6F, 16M; mean age = 20.9; all right-handed; 10 excluded | 35cm^2^ electrodes over O1/PO7 and O2/PO8 (tRNS) or over Oz and Cz (a-tDCS) | 1mA hf-tRNS (101-640Hz) with 0mA offset and 1mA a-tDCS for 20 min with a 15s fade-in/out | 5s tRNS fade-in/fade-out | Phosphene thresholds were significantly reduced after tRNS up to 60 min post stimulation relative to baseline, a behavioral marker of increased excitability of the visual cortex, while a-tDCS had no effect. |
| Qi et al., 2019 | Investigated whether execution-dependent motor cortex excitability is affected by prior interaction between tRNS and action observation. Measured single-pulse TMS-elicited MEPs with SI_1mV_ before action observation, immediately after, and after performing action execution. | 129 healthy volunteers; 81F, 48M; 24.42 ± 3.84; age range: 18–37; all right-handed | 5x5cm electrodes over left M1, and over contralateral supraorbital region | 1mA (peak-to-peak) hf-tRNS (101-640Hz) for 10 min with 5 s fade-in/out | 30s tRNS | Prior interaction between hf-tRNS and action observation of mirror-matched movements enhanced M1 excitability. The subsequent congruent goal-directed actions further enhanced the respective excitability alterations. |
| Parkin et al., 2019 | Investigated excitatory and inhibitory tES effects after stimulation delivered via unilateral versus bilateral electrode montage. Measured single-pulse TMS-elicited MEPs with SI_1mV_ before and after tES intervention execution. | 51 healthy volunteers; 32F, 19M; mean age: 20.6; age range: 18–27; all right-handed  Exp.1a: 8, Exp.1b: 9, Exp.1c: 9, Exp.2a: 17, Exp.2b: 8 | 5x7cm electrodes over left M1, and over contralateral orbit (unilateral) or right M1(bilateral) | 1mA unilateral or bilateral tDCS for 10 min with a 15s fade-in/out  1mA (peak-to-peak) unilateral or bilateral hf-tRNS (101-640Hz) with 0mA offset for 10 or 20 min with 20s fade-in/out | 2 electrodes montages | Effects of unilateral tES do not extend to the bilateral montage (for tRNS and tDCS) and to longer (double) stimulation duration (tRNS). |
| Moret et al., 2019 | Investigated whether both the lower and the higher half of the high-frequency band are needed for increasing neural excitability with tRNS.  Measured single-pulse TMS-elicited MEPs with SI_1mV_ before and after intervention. | 14 healthy volunteers; 14F, 0M; mean age 21 age range: 19–25; all right-handed | 16cm^2^ electrode over left M1, 60cm^2^ electrode over contralateral orbitofrontal area | 1.5 mA low-hf-tRNS (100–400 Hz), high-hf-tRNS (400–700 Hz), whole-hf-tRNS (100–700 Hz) with 0mA offset, for 10 min with 30s fade-in; intervals between sessions 1-3 days (and 2 months between experiments low/high and whole hf-tRNS) | 30s 1.5mA tRNS (15s fade-in/out) | Efficacy of hf-tRNS is related to the width of the selected frequency range. Only the full-high frequency band condition (100–700 Hz) modulated cortical excitability.  Neither the higher nor the lower sub-range of the high-frequency band significantly modulated cortical excitability. |
| Kortuem et al., 2019 | Investigated the effect of corticospinal excitability during sham stimulation on the individual response to tRNS and tACS.  Measured single-pulse TMS-elicited MEPs with SI_1mV_ before and after intervention. | 30 healthy volunteers; 10F, 20M; 24.2±2.8, age range: 18–30; all right-handed | 5x7cm electrodes over left M1, and over contralateral supraorbital area | 1mA tRNS (0.1-640Hz) and 140 Hz tACS for 10 min, with 5s fade-in/out; sessions  separated by at least 7 days | sham* | Individual responsiveness to sham stimulation can serve as a potential predictor of the variable effects of tRNS. Participants who did not exhibit any modulation of MEP amplitude after sham stimulation showed effects of active tRNS. In contrast, those who respond to sham (in both directions) did not present any response in the verum stimulation condition |
| Schoisswohl et al., 2021 | Investigated the effects of high- and low-frequency tRNS on excitability of AC.  Measured ASSR of 20 and 40Hz stimuli as well as power of oscillatory brain activity using EEG before and after tRNS. | 22 healthy volunteers; 11F, 11M; 24.18±2.89; age range: 19–31 | 5x7cm electrodes over the left (FT7, FC5, C5, T7, CP5, TP7) and the right hemisphere (FT8, FC6, C6, T8, CP6, TP8); both oriented in anterior-posterior direction | 2mA lf-tRNS (0.1-100Hz) and hf-tRNS (101-640Hz) for 20 min with 10s fade-in/out | 10s fade-in/out | Stimulation of both verum tRNS protocols revealed no significant changes either in ASSR or in resting state EEG activity. Sham tRNS resulted in a significant decrease in 20Hz ASSR and an increase in the alpha frequency band (8–12.5Hz). |
| Zhang et al., 2021 | Investigated the effects and optimal stimulation parameters of tACS and tRNS for modulating excitability of human pharyngeal motor cortex. Measured single-pulse TMS-elicited pharyngeal MEPs and thenar MEPs before and after intervention. | 15 healthy volunteers; 9F, 6M; 24±8; age range: 18–50 | 5x7cm electrodes over the “pharyngeal” area of M1 assessed with TMS and over contralateral supraorbital ridge | 1.5mA (peak-to-peak) tACS (10Hz alpha, 20Hz beta, and 70Hz gamma) and tRNS (0.1–640Hz) for 10 min with 10s fade-in/out | 10s of 20Hz tACS | Both gamma tACS and tRNS enhanced human pharyngeal cortical excitability. A significant MEP interaction was found both in the stimulated pharyngeal cortex and in the ipsilateral thenar cortex. Compared to sham, subsequent post hoc tests showed site-specific and sustained (60–120 min) increases in pharyngeal MEPs with tRNS and gamma tACS, and for thenar MEPs with beta tACS. |
| **1.2 Acute online effects during tRNS** | | | | | | |
| Potok et al., 2021 | Investigated the influence of tRNS on cortical responsiveness.  Measured RMT and probability of single-pulse TMS-elicited MEPs during brief tRNS delivery. | 81 healthy participants, 46F, 35M; 25.5±5; age range: 18-46, all right-handed  Exp.1: 16 (9F, 7M, 24.7±5, age range: 19–35);  Exp.2: 22 (13F, 9M; 25.4±5.4; age range: 20–46);  Exp.3: 20 (10F, 10M; 27.5±6; age range: 28–42);  Exp.4: 23 (14F, 9M; 24.3±3.9; age range: 19–34) | Exp.1 and 2: 5x7cm electrodes over left and right M1  Exp.3 and 4: 5x7cm electrodes placed ±7cm anterior and posterior to the left M1 along the coil axis (45º away from the nasion-inion mid-sagittal line) | 0.5-2mA (peak-to-baseline) hf-tRNS (100-500Hz) with 0mA offset for 3 s per trial with 0s fade-in/out vs no tRNS or active control (randomly interleaved) | no tRNS or active control condition (2mA peak-to-baseline hf-tRNS for 3s per trial) | tRNS acutely modulates the responsiveness of neural circuits of human M1 reflected in the immediate decrease in RMT and increase in probability of eliciting MEP for subthreshold TMS. |
| **2. Behavioural effects** | | | | | | |
| **2.1 Offline after- and learning effects following tRNS** | | | | | | |
| **2.1.1 Visual perception** | | | | | | |
| Fertonani et al., 2011 | Investigated the possibility of inducing differential plasticity effects using tDCS and tRNS during visual perceptual learning training as measured with orientation discrimination task performance. | 99 healthy volunteers; all right-handed; normal or corrected to normal vision  Pilot:  6 participants, 4F, 2M; 35.0±7.2; age range 29–48;  Main Exp.: 84 participants 42 males, mean age 21.7±2.5; age range 19-30; 6 groups x14sbj; Control hf-tRNS Exp.: 9 participants, 6F 3M, 31.7±3.9; age range 24–38 | 16cm^2^ electrode over occipital cortex (3.5 ± 0.2 cm above the inion) or over Cz (active control), and 60cm^2^ electrode on the right arm | 1.5mA hf-tRNS (100 – 640 Hz), lf-tRNS (0.1–100 Hz) with 0mA offset, a-tDCS, c-tDCS for 4 min x 5 first blocks (~22 min total) | 20s stimulation and active control condition | tRNS modulates learning effects during orientation discrimination task execution.  hf-tRNS significantly improved performance accuracy compared with a-tDCS, c-tDCS, sham, and active control site stimulations. |
| Pirulli et al., 2013 | Investigated how different types of tES (tDCS and tRNS) can modulate behavioral performance in the healthy adult brain in relation to their timing of application. Two protocols tested: before (offline) or during (online) visual perceptual learning training. Measured orientation discrimination task performance. | 90 healthy volunteers 45F, 45M, 21.8±2.9, age range: 19-32; all right-handed; normal or corrected to normal vision; 6 groups (online stimulation data from Fertonani 2011) | 16cm^2^electrode over occipital cortex V1 (3.5 ± 0.2 cm above the inion), 60cm^2^electrode on the right arm | 1.5mA offline-hf-tRNS (101 – 640 Hz)  online-hf-tRNS (101 – 640 Hz) with 0mA offset,  offline-a-tDCS  online-a-tDCS  for 4 min x 5 blocks (~total 20 min) | 20s tDCS  online-sham  offline-sham | Timing of tES protocols yields opposite effects on performance. tRNS facilitated task performance only when it was applied during task execution, whereas anodal tDCS induced a larger facilitation if it was applied before task execution. |
| Pirulli et al., 2016 | Investigated differences in visual perceptual learning caused by the position of the so-called reference electrode relative to the active electrode.  Measured orientation discrimination task performance before and after tRNS, tRNS-reversed, or sham. | 33 healthy volunteers; 20 F, 13 M; 25.1±5.5; 11 per group; | 16cm^2^ electrode over occipital lobe, 60cm^2^ electrode over the right upper arm (in the tRNS-reversed condition the cable connections with the stimulator were reversed) | 1.5 mA (peak-to-peak) hf-tRNS (100-640 Hz) for 4 min x 5 blocks | sham* | tRNS over the occipital cortex improved subject performance in the orientation discrimination task irrespective of the electrode configuration used. Effects of both tRNS and tRNS-reversed were different from sham, but not different from each other. |
| Contemori et al., 2019 | Investigated the effect of tRNS on perceptual learning (peripheral crowding task) and transfer in peripheral high-level visual tasks measured before and after intervention. | 32 healthy volunteers; 17F, 15M; mean age 25, age range: 20-32; normal or corrected to normal vision | 16cm^2^ electrode over occipital cortex (3cm above the inion), 27cm^2^ electrode over the vertex | 1.5mA (amplitude) hf-tRNS (100-640Hz) with 0mA offset for 30 min | 15s fade-in/out | Coupling tRNS to the early visual cortex with perceptual learning of a peripheral crowding reduction task is effective in boosting between-session learning but does not increase transfer of learning to untrained visual functions with respect to perceptual learning alone.  After training, the tRNS group showed greater learning rate (decrease in crowding threshold) with respect to the sham group. For both groups, learning generalized to the same extent to the untrained retinal location and task. |
| Fertonani et al., 2019 | Investigated tRNS and a-tDCS of the V1 during visual perceptual learning in healthy young and older individuals.  Measured orientation discrimination task performance and TEP over V1 before and after intervention. | 45 young participants; 22F, 23M; 22.3±3.1 with normal or corrected-to-normal vision.  36 older participants; 21F, 15M; 66.1±3.6; with normal or corrected-to-normal vision  3 stimulation groups | 16cm^2^ electrode over Oz; 60cm^2^ electrode over right shoulder | 1.5mA a-tDCS and 1.5mA hf-tRNS (101-600Hz) with 0mA offset for 22 min | 20s tES at the beginning and at the end | Only the tRNS in the young, but not in the older, subjects modulated visual perceptual learning, by decreasing performance. TEP-revealed age-related changes in connectivity, that is, a stronger activation of the prefrontal cortex after visual cortex stimulation, and a stronger modulation of the prefrontal cortex after visual perceptual learning in the older subjects. |
| Herpich et al., 2019 | Investigated the effect of tRNS on visual perceptual learning in intact and brain-damaged humans. Tested whether tRNS of V1 during training can enhance and speed up the resultant perceptual learning.  Measured motion discrimination task performance. | 45 healthy volunteers; 32 F, 13M; mean age: 19, age range: 19–36; normal or corrected to normal vision; all right-handed; 15 per group  11 patients with cortical blindness; normal or corrected to normal visual acuity; all right-handed; 3 tRNS, 2 sham, 6 only training | tRNS: 35cm^2^ electrodes positioned bilaterally over O1 and O2  a-tDCS: 35cm^2^ electrodes over Oz and Cz active control tRNS: 35cm^2^ electrodes positioned bilaterally over P3 and P4 | 1mA hf-tRNS (101-640Hz) or a-tDCS  For 20 min with 20s of fade-in/out, 10 days of training and follow-up after 6 months | sham group: 20s tRNS; no stimulation condition; active control condition | Enhancement of the capacity for long-lasting plastic and restorative changes when a neuromodulatory intervention is coupled with visual training (motion perception).  Relative to control conditions and anodal stimulation, tRNS-enhanced learning was at least twice as fast, and, crucially, it persisted for 6 months after the end of training and stimulation. Notably, tRNS also boosted learning in patients with chronic cortical blindness, leading to recovery of motion processing in the blind field after just 10 days of training, a period too short to elicit enhancements with training alone. |
| Ghin et al., 2021 | Investigated the spatial and temporal dynamics of cortical activity modulated by offline hf-tRNS on performance of a motion direction discrimination 2IFC task.  Measured amplitude of motion-related VEPs over the parieto-occipital cortex, oscillatory PSD at rest, as well as shift in ERSP in response to the motion stimuli between the pre- and post-stimulation period, using EEG. | 16 healthy volunteers; 9F, 7M; age range 19–33 all right-handed | 16cm^2^ electrodes over PO3 and PO4, bilaterally over the parieto-occipital cortex | 1.5mA hf-tRNS (100-600Hz) with 0mA offset for 20 min | 30s 1.5mA tRNS | Offline hf-tRNS may induce moderate after-effects in brain oscillatory activity but not behavioral task performance. The accuracy of the motion direction discrimination task was not modulated by offline hf-tRNS. Although the motion task was able to elicit motion dependent VEP components (P1, N2, and P2), none of them showed any significant change between pre- and post-stimulation. There was a time-dependent increase in the PSD in alpha and beta bands regardless of the stimulation protocol. The time–frequency analysis showed a modulation of ERSP power in the hf-tRNS condition for gamma activity when compared to pre-stimulation periods and Sham stimulation. |
| **2.1.2 Somatosensory perception** | | | | | | |
| Saito et al., 2019 | Investigated the effects of tES applied to primary somatosensory cortex on SEP-PPD and tactile discrimination performance. Measured N20/P25_SEP-PPD, N20_SEP-PPD, and P25_SEP-PPD responses and grating orientation task performance assessed before and immediately after tES applied to primary somatosensory cortex. | 17 healthy volunteers; 0F, 17M; 22.0±1.1; 16 right-handed | 5x5cm electrodes located 3 cm posterior to C3 (left primary somatosensory cortex), and over the contralateral orbit | 0.7mA a-tDCS, a-tPCS (50ms pulse, 5ms IPI), tACS (140Hz, Exp.1), tRNS (0.1-640Hz), 0mA offset, for 10 min with 10s fade-in/out  4 (or 3 in Exp.2) sessions with 3 days break | 30s tDCS | tRNS and anodal tPCS can improve sensory perception by modulating neuronal activity in primary somatosensory cortex.  a-tDCS and a-tPCS decreased N20_SEP-PPD, and tRNS increased the first N20 SEP amplitude.  tRNS and a-tPCS improved grating orientation task performance, reflected in decreased discrimination threshold. |
| **2.1.3 Motor function** | | | | | | |
| Chaieb et al., 2009 | Measured BOLD fMRI to monitor modulations in human sensorimotor activity (activation maps for a right-hand index–thumb finger-tapping task) after the application of 4-min tRNS. | 9 healthy volunteers; 3F, 6M; age range: 21-32; all right-handed | 4x4cm electrode over sensorimotor cortex, 6x14cm electrode over contralateral orbit | 1mA tRNS (0.1-640Hz) with 0mA offset for 4 min | sham* | Short-duration application of tRNS can induce a transient decrease in BOLD activity in the human primary sensorimotor cortex, using a classical finger-tapping task. |
| Saiote et al., 2013 | Investigated the effects of tDCS and tRNS in the early and later stages of visuomotor learning, as well as associated brain activity changes using fMRI (throughout the experiment).  Measured motor task performance changes after the stimulation. | 52 healthy volunteers; 30F, 22M; 27±6; age range: 20-50; all right-handed  5 groups of 10 people  a-tDCS, c-tDCS, lf-tRNS, hf-tRNS, sham | 5x7cm MRI compatible electrodes over left M1 and over contralateral right orbit | 1mA a-tDCS, c-tDCS,  lf-tRNS (0.1-100Hz) or hf-tRNS (101-640Hz)  for 10 min of a task with 20s fade-in and 10s fade-out | 20s fade-in and 10s fade-out | lf-tRNS and hf-tRNS differentially modulate visuomotor learning. Cathodal tDCS and hf-tRNS showed a tendency to improve and lf-tRNS to hinder early learning during stimulation, an effect that remained for 20 minutes after cessation of stimulation in the late learning phase. Motor learning-related activity decreased in several regions, however, there was no significant modulation of brain activity by tDCS. hf-tRNS was associated with reduced motor task-related-activity bilaterally in the frontal cortex and precuneous, probably due to interaction with ongoing neuronal oscillations. |
| Prichard et al., 2014 | Investigated whether tDCS and tRNS alter aspects of learning a tracing task: skill acquisition (online/within session effects) or consolidation (offline/between session effects). | 91 healthy volunteers; 52F, 39M; 25.7±4.6; all right-handed  5 groups with 18 participants | 16cm^2^ electrodes over: tDCS and tRNS: M1 and contralateral supraorbital area; tDCS: left and right M1; tRNS: right T6 and contralateral supraorbital area (control) | 1mA tDCS or hf-tRNS (100-640Hz)  For 20 min with 15s fade-in/out; during the task  3 consecutive days | 30s tRNS or tDCS | Unilateral M1 stimulation using tRNS as well as unilateral and bilateral M1 tDCS all enhanced motor skill learning compared to sham stimulation. In all groups, this appeared to be driven by online effects without an additional offline effect. Unilateral tDCS resulted in large skill gains immediately following the onset of stimulation, while tRNS exerted more gradual effects. Control stimulation of the right temporal lobe did not enhance skill learning relative to sham. |
| Abe et al., 2019 | Investigated the effects of tRNS on both corticospinal excitability and motor performance.  Measured single-pulse TMS-elicited MEPs with SI_1mV_ and performance of visuomotor tracking task by isometric abduction motion of the right index finger before and after intervention. | 16 healthy volunteers; 4F, 12M; 21±0.35; all right-handed | 5x7cm electrodes over left M1, and over right front forehead area | 1mA of tRNS (0.1-640Hz) for 10 min with 10s fade-in/out  tRNS vs sham separated by 1 week | 30s tRNS | tRNS over M1 is effective for enhancing cortical excitability as well as for motor performance. Significant increase in MEP amplitudes immediately and 10 min after tRNS, motor performance improved 10 min after tRNS. |
| De Albuquerque et al., 2019 | Investigated the influence of tRNS on motor skill acquisition and retention in a complex golf putting task.  Measured task performance before, during and after intervention. | 34 healthy volunteers, 0F, 34M; 23.1 ± 2.8; age range: 18-30, all right-handed  tRNS vs  sham group | 7x5cm electrodes over M1 and over the contralateral supraorbital region | 2mA hf-tRNS for 20min during task performance | 30s tRNS with 10s fade-in/out | Acute application of tRNS failed to enhance skill acquisition or retention in a golf putting task – no difference between tRNS and sham groups |
| Hoshi et al., 2021 | Investigated the effects of tRNS timing on corticospinal excitability and motor function when combined with motor training.  Measured single-pulse TMS-elicited MEPs with SI_1mV_ and visuomotor tracking task performance before and after tRNS and motor training. | 15 healthy volunteers; 0F, 15M; 21.32 ± 0.58; all right-handed | 5x7cm electrodes over left M1, and over contralateral orbital area | 1mA tRNS (0.1–640 Hz) for 10 min, with 10s fade-in/out, before, during or after motor training.  Crossover design with 4 conditions randomly performed with a break of at least one week between each condition. | 30s tRNS | The timing of tRNS affects corticospinal excitability but not motor learning.  The corticospinal excitability increased after tRNS in the before and during conditions but not in the after condition. Motor function after motor training improved in all conditions, but there were no significant differences between these conditions. |
| **2.1.4 Clinical: visual perception** | | | | | | |
| Camilleri et al., 2014 | Investigated whether a short behavioral training using a contrast detection task combined with online tRNS was as effective in improving visual functions in participants with mild myopia compared to a 2-month behavioral training without tRNS. Measured UCVA and UCCS before and after intervention using Landolt-C and Grating tests of the FrACT. | 16 volunteers with mild myopia, mean age 24.12, age range: 19 and 27  2 groups of 8 people tRNS+training vs training only | 16cm^2^ electrode over occipital cortex, 3cm above the inion, 60cm^2^ electrode over upper right arm | 1.5mA hf-tRNS (100-640Hz) with 0mA offset for 5min during 5 first training blocks (total 25min stimulation)  8 sessions | training without tRNS | The combination of behavioural training and tRNS can be fast and efficacious in improving sight in individuals with mild myopia. After 2 weeks of perceptual training in combination with tRNS, participants showed an improvement of 0.15 LogMAR in UCVA that was comparable with that obtained after 8 weeks of training with no tRNS, and an improvement in UCCS at various spatial frequencies (whereas no UCCS improvement was seen after 8 weeks of training with no tRNS). A control group that trained for 2 weeks without stimulation did not show any significant UCVA or UCCS improvement. |
| Campana et al., 2014 | Investigated whether shorter perceptual training combined with hf-tRNS can improve visual functions in a group of adult participants with amblyopia.  Measured VA and CS function using Landolt-C and Grating tests of Freiburg Visual Acuity Test and CRS Psycho 2.36 test.  Pilot study. | 7 volunteers with anisometric amblyopia, mean age: 39.2, age range: 26-52 | 16cm^2^ electrode over occipital cortex, 3cm above the inion, 60cm^2^ electrode over forehead | 1.5mA hf-tRNS (100-640Hz) with 0mA offset for 5min during 5 first training blocks (total 25min stimulation)  8 sessions | none | Eight sessions of training in contrast detection under lateral masking conditions combined with hf-tRNS, were able to substantially improve VA and CS function in adults with amblyopia. |
| Camilleri et al., 2016 | Investigated the efficacy of a short training (8 sessions) using a single Gabor contrast-detection task with concurrent hf-tRNS in comparison with the same training with sham stimulation or hf-tRNS with no concurrent training, in improving VA and CS of individuals with uncorrected mild myopia.  Measured UCVA and UCCS using Landolt-C and Grating tests of FrACT before and after intervention | 30 volunteers with mild myopia, mean age 25.32, age range: 19 and 29  3 groups of 10 people  tRNS+training vs sham+training vs tRNS only | 16cm^2^ electrode over occipital cortex, 3cm above the inion, 60cm^2^ electrode over forehead | 1.5mA hf-tRNS (100-640Hz) with 0mA offset for 5min during 5 first training blocks (total 25min stimulation)  8 sessions | 20s tRNS at the beginning of each block | Hf-tRNS coupled with a short training of contrast detection task is able to improve VA and CS, whereas no effect on VA and marginal effects on CS are seen with the sole administration of hf-tRNS. |
| Moret et al., 2018 | Investigated the efficacy hf-tRNS combined with a short perceptual training (2IFC task contrast detection of a central Gabor patch flanked by two high-contrast collinear Gabors - lateral masking) in adults with amblyopia.  Measured VA and CS before and after intervention. | 20 volunteers with amblyopia, 12F, 8M, mean age 44, age range: 27 and 58  2 groups of 10 people  tRNS+ training vs sham+ training | 16cm^2^ electrode over occipital cortex, 3cm above the inion, 60cm^2^ electrode over forehead | 1.5mA hf-tRNS (100-640Hz) with 0mA offset for 5min with 30s fade-in during 5 first training blocks (total 25min stimulation)  8 sessions | 30s fade-in/out 1.5mA tRNS | Significant and similar improvement of CS for both groups, suggesting that hf-tRNS is not crucial for the improvement of CS. However, for VA, a significant improvement was only observed in the hf-tRNS group with a mean VA improvement of 0.19 LogMAR in the amblyopic eye after 8 sessions. |
| Donkor et al., 2021 | Investigated whether five daily sessions of tRNS over V1 would improve perceptual learning measured with CS, crowded and uncrowded VA in adults with amblyopia measured during and after stimulation. | 19 volunteers with amblyopia, 9F, 10M, 44.2±14.9  tRNS: 9  sham: 10 | 5x5cm electrodes over Oz, and over Cz | 2mA tRNS (0.1-640Hz) for 25min with 30s fade in/out over 5 consecutive days | 30s fade-in/out 2mA tRNS | tRNS induced short-term contrast sensitivity improvements in adult amblyopic eyes, and the effects may extend to uncrowded visual acuity. However, multiple sessions of tRNS did not lead to enhanced or long-lasting effects. |
| **2.1.5 Clinical: auditory perception** | | | | | | |
| Vanneste et al., 2013 | Investigated the efficacy of three different tES techniques: tDCS, tACS, and tRNS applied to AC in tinnitus patients. Measured tinnitus loudness and distress before and after intervention. | 111 tinnitus patients 77F, 34M, 49.46±14.37  tinnitus duration 4.18±4.05  tDCS (reversed placement): 16 and 20  tRNS: 38  tACS: 37 | 35cm^2^ electrodes over T3, and over T4 (AC bilaterally) | 1.5mA tDCS for 20min with 10s fade in  1.5mA tACS (at IAF peak within the range of 6–13 Hz) for 20min with 10s fade in  1.5mA lf-tRNS (0.1-100Hz) with 0mA offset for 20min with 10s fade in | none | Lf-tRNS induced the larger transient suppressive effect on tinnitus loudness and tinnitus related distress as compared to tDCS and tACS |
| Joos et al., 2015 | Investigated the efficacy of lf-tRNS, hf-tRNS and tRNS on non-pulsatile tinnitus.  Measured tinnitus loudness and distress before and after intervention. | 154 patients with non-pulsatile tinnitus, 30F, 124M; 53.28±12.11; tinnitus duration  6.92±6.64  lf-tRNS: 119  hf-tRNS: 19  tRNS: 16 | 35cm^2^ electrodes over T3, and over T4 | 2mA lf-tRNS (0.1-100Hz), hf-tRNS (100-640Hz) and tRNS (0.1-640Hz) with 0mA offset for 20min with 10s fade in | none | Reduction in tinnitus loudness when lf-tRNS and hf-tRNS were applied as well as a reduction in tinnitus-related distress with lf-tRNS. Significantly more pronounced reduction in loudness and distress in pure tone tinnitus compared to narrow band noise tinnitus when hf-tRNS was applied, a difference that could not be obtained with lf-tRNS. |
| To et al., 2017 | Investigated the effects of treatment protocol using multisite tES over tinnitus network (combined bifrontal tDCS and bilateral tRNS) on tinnitus intensity and distress.  Measured tinnitus loudness and distress before and after intervention. | 40 patients with tinnitus 18F, 22M, 48.33±10.74; tinnitus duration 10.82±14.35  3 groups: tDCS: 12  tDCS+tRNS: 14  waiting list: 14 | 35cm^2^ electrode over left DLPFC (c-tDCS), and over right DLPFC (a-tDCS)  35cm^2^ electrode over T3 and T4 (tRNS) | 1.5mA tDCS for 20 min with 10s fade in  2mA lf-tRNS (0.1-100Hz) with 0mA offset for 20min with 10s fade in  8 sessions (2x per week for 4 weeks)  Waiting list – no treatment | no stimulation group | Multisite treatment tES protocol resulted in more pronounced effects when compared with the bifrontal tDCS protocol or the waiting list group, suggesting an added value of auditory cortex tRNS to the bifrontal tDCS protocol for tinnitus patients. |
| Mohsen et al., 2018 | Investigated the multisite protocol of tRNS by applying lf-tRNS over the AC preceded by hf-tRNS over the right DLPFC in a sham-controlled clinical trial and compare the results to the auditory lf-tRNS.  Measured tinnitus loudness and annoyance before and after intervention. | 32 patients with tinnitus 9F, 23M, 42±10.96  AC: 16  DLPFC+AC: 16  Each group sham 30min break and real session | 35cm^2^ electrodes over F4 and FP1 (DLPFC)  35cm^2^ electrodes over T3 and T4 (AC) | 2mA lf-tRNS (0.1-100Hz) for 20min  For multisite protocol 10 min over PFC followed by 10 min over AC | sham* | The multisite tES protocol was more effective in reducing the loudness and annoyance of tinnitus in comparison with auditory cortex tRNS, while the sham stimulation session had no effect. |
| Mohsen et al., 2019b | Investigated the role of applying eight sessions of multisite tRNS in decreasing tinnitus loudness and annoyance without exerting additional adverse effects.  Measured tinnitus loudness and annoyance before and after intervention. | 29 patients with tinnitus 8F, 21M, 45.34±9.57  1 session: 17  8 sessions: 12 | 35cm^2^ electrodes over F4 and FP1 (DLPFC)  35cm^2^ electrodes over T3 and T4 (AC) | 2mA lf-tRNS (0.1-100Hz) over AC and hf-tRNS (100-640Hz) over DLPFC with 0mA offset for 10 min over PFC followed by 10 min over AC with 30s fade-in/out | none | Statistically and clinically significant reduction in tinnitus loudness and annoyance in both groups, while the amount of annoyance suppression in the multiple-sessions group was significantly greater than the single-session group. The patients of the multiple session tRNS group reported an improvement in their sleep and lower tinnitus handicap inventory scores without experiencing any additional adverse effects of the intervention. |
| Mohsen et al., 2019a | Investigated the modulatory effects of multisite tRNS on the tinnitus network. EEG recorded before and after the session. | 32 patients with tinnitus 9F, 23M, 42±10.96  AC: 16  DLPFC+AC: 16  Each group sham 30min break and real session | 35cm^2^ electrodes over F4 and FP1 (DLPFC)  35cm^2^ electrodes over T3 and T4 (AC) | 2mA lf-tRNS (0.1-100Hz) for 20min  For multisite protocol 10 min over PFC followed by 10 min over AC | 10s tRNS | Increased power in the alpha-1 band at the AC and PFC accompanied by decreased power in the delta and beta-2 bands in the PFC after the multisite-tRNS real session.  Standardized low-resolution brain electromagnetic tomography (sLORETA) showed a significant decrease in beta-2 activity in the PFC, ACC, and the paraHC and decreased alpha connectivity between the right PFC and the left AC. No significant effects were observed for the sham session. |
| Kreuzer et al., 2019 | Investigated the use of hf-tRNS in a one-arm pilot study in patients with chronic tinnitus. Measured primary (treatment response - tinnitus questionnaire) and secondary outcomes (tinnitus numeric rating scales, depressivity, and quality of life) before and after intervention.  Pilot study. | 30 patients with tinnitus 4F, 26M, 49.2±10.9  tinnitus duration: 96.0±73.7 months  who underwent rTMS treatment before | 5x7cm electrodes over T7, and over T8 | 2mA hf-tRNS (100-640Hz) with 0mA offset for 20 min with 10s fade-in/out  10 sessions (2 weeks Mon-Fri) | none | hf-tRNS is feasible for daily treatment in chronic tinnitus. However, summarizing low treatment response, increase of tinnitus loudness in 20% of patients and missing of any significant secondary outcome, the use of hf-tRNS as a general treatment for chronic tinnitus cannot be recommended at this stage. Differences in treatment responders between tRNS and rTMS highlight the need for individualized treatment procedures. |
| **2.1.6 Clinical: pain and motor function** | | | | | | |
| Stephani et al., 2011 | Investigated stimulation-induced cortical plasticity of iTBS and tRNS in patients with Parkinson’s disease. Single-pulse TMS-elicited MEP with SI_1mV_ measured before and after intervention. | 8 non-tremor-dominant idiopathic Parkinson’s disease patients; 1F, 7M; 62.23±8.3 | 16cm^2^ electrode over abductor digiti minimi muscle hotspot and 35cm^2^ electrode over contralateral orbital region | iTBS with 80% of RMT or 1mA (peak-to-peak) tRNS (0.1-640Hz) for 10 min | sham iTBS with shielded figure-of-eight coil | Decrease in MEP amplitude after tRNS. No statistical significance for the factor of time or interaction. Anti-parkinsonian drugs were not discontinued in the study and dopaminergic drugs may have contributed to the paradoxic effects of tRNS. No “off” medication control group. |
| Palm et al., 2016 | Investigated the effects of tRNS over DLPFC on attention and neuropathic pain in Multiple Sclerosis patients. Measured pain, attention, mood and electrophysiological parameters (EEG). | 16 Multiple Sclerosis patients; age range: 18-70 years; right-handed | 25cm^2^ electrodes over F3, and over AF8 | 2mA (peak-to-peak) tRNS (0-500Hz) with 1mA DC offset  Two blocks (3 consecutive daily sessions) tRNS/sham separated by 3-week wash-out interval | 15s fade-in tRNS | Compared to sham, tRNS showed a trend to decrease the N2-P2 amplitudes of pain related evoked potentials and improve pain ratings. Attention performance and mood scales did not change after stimulations. |
| Salemi et al., 2019 | Investigated the effects of tRNS on fatigue in subjects with relapsing–remitting Multiple Sclerosis with low physical disability.  Pilot study | 17 Multiple Sclerosis patients; 9 received real tRNS, 6F, 2M,  8 received sham, 6F, 3M, | 5x5cm electrode over dominant M1 or contralateral to the most compromised limb; 5x5cm over C3 + FP2 or C4 + FP1 | 1.5mA hf-tRNS (100-640Hz) with 0mA offset for 15 min over two consecutive weeks (for 10 days) | 30s tRNS | In the tRNS group, beneficial effects were observed using the Modified Fatigue Impact Scale (physical subscale), the subscales ‘change in health’ and ‘role limitations due to physical problems’ of the Multiple Sclerosis Quality of Life-54, and by assessing the patient impression of perceived fatigue. |
| Arnao et al., 2019 | Investigated the combined use of tRNS with the Graded Repetitive Arm Supplementary Program in sub-acute ischemic stroke patients suffering from arm impairment.  Measured upper limb impairment with Fugl-Meyer Assessment–Upper extremity before and after treatment. Pilot study. | 18 ischemic stroke patients with upper limb disability, evaluated by FMA–UE,1–6 weeks after stroke  9 in experimental group, 5F, 4M, 75.5±11.7; 9 in control group, 4F, 5M, 76.6±6.6 | One electrode was placed over M1 opposite the upper limb impairment, and the reference electrode was placed over the contralateral orbit | 1mA hf-tRNS (101-640Hz) for 20 min on  5 sessions | 30s tRNS | Proposed protocol might have a positive impact on clinical rehabilitation programs, given it is effective, easy to carry out, well tolerated by the patient and can be initiated in the sub-acute phase bedside at a Stroke Unit. Beneficial effects in the tRNS group correlated with better Fugl-Meyer Assessment–Upper extremity score than sham stimulation group and these results did not correlate to stroke severity. |
| ﻿Monastero et al., 2020 | Investigated the effects of tRNS applied over M1 in Parkinson’s disease patients with mild cognitive impairment in cognitive and motor tasks. Measured cognitive and motor function before and after each session. | 10 Parkinson’s disease patients with mild cognitive impairment; 0F, 10M; 70.2±8.7; age range: 59-80 | 4.5x4.5cm electrodes over the left M1 and the contralateral shoulder | 1.5mA hf-tRNS (100-600Hz) for 15 min with 10s fade-in/out  Double-blind | 30s 1.5mA tRNS with 10s fade-in/out | tRNS improved the motor ability (measured with Unified Parkinson’s Disease Rating Scale) in comparison to sham control condition. No other significant differences were found in other motor tasks and cognitive assessment both after real and sham stimulations. |
| **2.2 Acute online effects during tRNS** | | | | | | |
| **2.2.1 Visual perception** | | | | | | |
| Campana et al., 2016 | Investigated the effects of hf- vs lf-tRNS on motion adaptation and recovery employing motion after-effect phenomenon.  Measured the estimated motion after-effect duration during tRNS stimulation. | 36 healthy volunteers; normal or corrected-to-normal vision  Exp.1  hf-tRNS: 12  lf-tRNS: 12  control: 12 | 25cm^2^ electrodes placed bilaterally over a site located ~3 cm above the inion and ~5 cm anteriorly on the left and on the right (hMT^+^) or over frontal areas | 1.5mA lf-tRNS (0.1-100Hz), hf-tRNS (100-640Hz) with 0mA offset for 17-18 min with 30s fade-in  sham on the same session | 30s fade in/out 1.5mA tRNS,  Active control condition | Hf- and lf-tRNS have opposite effects on the adaptation-dependent imbalance between neurons tuned to opposite motion directions.  When applied to the bilateral hMT+, hf-tRNS caused a significant decrease in motion after-effect duration whereas lf-tRNS caused a significant corresponding increase in motion after-effect duration. No effects on motion after-effect duration were induced by stimulating frontal areas. |
| van der Groen and Wenderoth, 2016 | Investigated whether noise added directly to cortical networks acutely enhances sensory detection. Tested the hypothesis that SR phenomenon underlies the tRNS mechanism of action.  Measured visual contrast detection 2AFC task performance during tRNS. | 52 healthy volunteers; 26F, 26M; mean age 24; age range: 18-30 all right-handed; normal or corrected to normal vision  Exp.1: 31 (14F, 17M; 24; age range: 19 –30)  Exp.2: 38 (21F, 17M; 25; age range: 19 –30)  Exp.3: 20 (9F, 11M; 25; age range: 21–30)  2 groups in each | 5x7cm electrodes over Oz, and over Cz in Exp.2  5x7cm electrodes over forehead (Fpz), and over Cz in Exp.3 | 0.5, 0.75, 1 or 1.5mA (peak-to-baseline) hf-tRNS (100-640Hz) with 0mA offset for 2.04 s twice per trial, randomized order | no tRNS or active control condition | When the optimal level of noise was added to V1, detection performance improved significantly relative to a zero-noise condition and to a similar extent as optimal noise added to the visual stimuli. Results demonstrate that adding noise to cortical networks can improve human behaviour and that tRNS is an appropriate tool to exploit the mechanism of SR. |
| Mcintosh and Mehring, 2017 | Investigated modulation of perceptual decisions with conflicting biases by applying tRNS.  Measured performance of a Simon task, a paradigm where irrelevant spatial cues influence the response times of subjects to relevant colour cues, measured during tRNS. Utilized DDM framework to analyse the data. | 24 healthy volunteers; 7F, 16M; age range: 20-40; all right-handed | 7x5cm electrodes over FT7 and FT8 | 1mA (peak-to-peak) tRNS (0.1-640Hz) for 18 first trial within each block (alternating with sham) first and last trial to ramp up and down  (18 trials tRNS + 18 trials sham) x 6 blocks x 4 sessions | sham* | Non-specific to the Simon task tRNS-induced reduction in the response time of subjects independent of the congruence between spatial and colour cues, but dependent on the baseline response time (tRNS reduces response time particularly when baseline response times are long). Different baseline responses resulted from interaction of noise with models of evidence accumulation. |
| Ghin et al., 2018 | Investigated the effects of hf-tRNS to those of a-tDCS and c-tDCS in a global motion direction discrimination task. An equivalent noise paradigm was used to assess how hf-tRNS modulates the mechanisms underlying local and global motion processing.  Measured motion coherence threshold and slope of the psychometric function using an 8AFC task in which observers had to discriminate the motion direction of a random dot kinematogram presented either in the left or right visual hemi-field. | 53 healthy volunteers; all right-handed; normal or corrected to normal vision  Exp.1: 16,  Exp.2: 24  Exp.3: 13 | Exp.1 and 3 16cm^2^ electrode over left hMT^+^  (3 cm dorsal to inion and 5 cm leftward) 60cm^2^ electrode over Cz  Exp.2a: 16cm^2^ electrode over Cz 60cm^2^ electrode over the left forehead  Exp.2b: 16cm^2^ electrode over Cz  60cm^2^ electrode over the left V1 (i.e., 3 cm dorsal to the inion and 1 cm leftward) | 1.5mA (peak-to-peak) hf-tRNS (100-600Hz) with 0mA offset  1.5mA a-tDCS and c-tDCS for 18 min | 30s stimulation | hf-tRNS interacts with the output neurons tuned to directions near to the directional signal, incrementing the signal-to-noise ratio and the pooling of local motion cues and thus increasing the sensitivity for global moving stimuli. hf-tRNS reduced the motion coherence threshold but did not affect the slope of the psychometric function, suggesting no modulation of stimulus discriminability. Anodal and cathodal tDCS did not produce any modulatory effects. Equivalent noise analysis in the last experiment found that hf-tRNS modulates sampling but not internal noise, suggesting that hf-tRNS modulates the integration of local motion cues. |
| van der Groen et al., 2018 | Investigated whether perceptual decisions made by human observers obey the SR principles, by adding noise directly to the visual cortex using tRNS while participants judged the direction of coherent motion in random dot kinematograms presented at the fovea.  Measured random dot motion task performance during tRNS. Utilized DDM to analyse the data. | 45 healthy volunteers; 17F, 28M; mean age 22.5; age range: 18-27 all with normal or corrected to normal vision  15 per group (bilateral, unilateral left and right) | Exp.1 bilateral: 4x4cm electrodes placed 3.5 cm above the inion and 6.5 cm left and right of the midline in the sagittal plane  Exp.2 unilateral left:  4x4cm electrodes placed over left V1 (as in Exp.1) and Cz  Exp.3 unilateral right:  4x4cm electrodes placed over right V1 (as in Exp.1) and Cz | 0.25, 0.375, 0.5 and 0.75mA hf-tRNS (100-640 Hz) with 0mA offset during 20 trials followed by 20 no stimulation trials within each block of 6min randomized order | no tRNS; 3 electrodes montages | Found that adding tRNS bilaterally to visual cortex enhanced decision-making when stimuli were just below perceptual threshold, but not when they were well below or above threshold. Bilateral tRNS selectively increased the drift rate parameter, which indexes the rate of evidence accumulation. |
| Battaglini et al., 2019 | Investigated whether inhibitory/facilitatory contrast sensitivity effects related to lateral masking are modulated by tRNS.  Measured contrast detection task performance.  Signal detection theory was used to measure sensitivity (d’) and the criterion in a detection task. | 68 healthy volunteers, 46F, 24M; 24 ± 3; with normal or corrected to normal vision  Exp.1: 19  Exp.2: 19  Exp.3: 30  Exp.4: 15 | 5x7cm electrodes over Oz and Cz (Exp.1 and 2) or over the forehead (between Fpz and nasion) and Cz (Exp.3 and 4) | 1.5mA hf-tRNS (100-600Hz) with 0mA offset for 12 min with 15s fade-in/out | 30s tRNS with 15s fade-in/out | Occipital stimulation results in a tRNS-dependent increased sensitivity for the single Gabor signal of low but not high contrast. Dissociation of the tRNS effects when the Gabor signal is presented with the flankers, consisting in a general increased sensitivity at 2λ where the flankers had an inhibitory effect (reduction of inhibition) and a decreased sensitivity at 6λ where the flankers had a facilitatory effect on the Gabor signal (reduction of facilitation). After a frontal stimulation, no specific effect of tRNS was found. |
| Pavan et al., 2019 | Investigated whether modulatory effects of hf-tRNS rely on the SR phenomenon, and what is the specific neural mechanism producing SR.  Measured performance of 2AFC motion direction discrimination task with a coherence near threshold measured during intervention. | Exp.1: 24 healthy volunteers; 13F, 11M; age range: 18-40; all right-handed; with normal or corrected to normal vision  12 in Exp.1A and 12 in Exp.1B;  Exp.2: 24 healthy volunteers; 15F, 9M; age range: 18-40; all right-handed; with normal or corrected to normal vision  12 in tRNS and 12 in sham  Exp.3: 20 healthy volunteers; 10F, 10M; age range: 18-40  4 groups of 5 participants | 16cm^2^ electrodes over left and right hMT^+^  (3 cm dorsal to inion and 5 cm leftward and rightward from there for the localization of the hMT^+^) | 0.5, 0.75, 1.0, 1.5, and 2.25mA hf-tRNS (100-600Hz) with 0mA offset, for 20 min  sessions on different, non-consecutive days | 30s 1.5mA tRNS; 30s 2.25mA tRNS; | The results showed a significant improvement in performance when hf-tRNS was applied at 1.5mA, representing the optimal level of external noise. However, stimulation intensity at 2.25mA significantly impaired direction discrimination performance. An equivalent noise analysis, used to assess how hf-tRNS modulates the mechanisms underlying global motion processing, showed an increment in motion signal integration (increase in sampling level) with the optimal current intensity, but reduced motion signal integration at 2.25 mA. |
| van der Groen et al., 2019 | Investigated the effect of noise on perceptual dominance durations.  Used a computational model and compared the model prediction to measured binocular rivalry dynamics when noise was added either to the visual stimulus or directly to V1 by tRNS. | 50 healthy volunteers; mean age 23.5 with normal or corrected to normal vision  Exp.1 peripheral noise: low contrast: 10, high contrast: 10  Exp.2 tRNS: low contrast: 15, high contrast: 15 | 5x7cm electrodes over Oz, and over Cz | 1mA hf-tRNS (100-640Hz) with 0mA offset for 5 s per trial tRNS/no tRNS randomized (total 6 min stimulation) | no tRNS | Adding noise significantly reduced  dominance duration of the mixed percept for low contrast visual stimuli for noise delivered both to the stimuli and to the cortex. Both central and peripheral noise can influence state-switching dynamics of binocular rivalry under specific conditions (e.g., low visual contrast stimuli), in line with a SR-mechanism. |
| Battaglini et al., 2020 | Investigated tRNS effects on multiple spatial frequencies and orientation to unravel whether the long-term perceptual improvements are due to early-stage perceptual enhancements of contrast sensitivity or later stage mechanisms such as learning consolidation.  Measured visual contrast detection task performance. | 20 healthy volunteers, 13F, 7M; 25 ± 3.4; age range: 21-31; with normal or corrected to normal vision | 7.2x6cm electrode over Oz, 11.5x9.5cm electrode over Cz | 1.5mA hf-tRNS (100-600Hz) with 0mA offset for 15 min with 15s fade-in/out  4 session each participant (tRNS vs. Sham) × Gabor patch orientations (vertical vs. diagonal) in 2 days; 30min break between sessions | 30s tRNS with 15s fade-in/out | Online tRNS effects on visual perception are the result of a complex interaction between stimulus intensity and cortical anatomy. tRNS enhances detection of a low contrast Gabor, but only for oblique orientation and high spatial frequency. No improvement was observed for low contrast and vertical stimuli. |
| Melnick et al., 2020 | Investigated underlying mechanisms of visual improvements caused  by tRNS using equivalent noise approach. Measured visual orientation discrimination task performance during and after intervention. | 10 healthy volunteers, 7F, 3M; age range: 18-32; with normal or corrected to normal vision; 1 excluded | 5x7cm electrodes bilaterally over occipital cortex at O1 and O2 | 2mA hf-tRNS (100-600Hz) during 400 trials of the task for ~ 20 min  3 sessions: baseline, tRNS and sham (the last 2 counterbalanced) | no tRNS | tRNS improves visual processing when stimulation is applied during task performance, but only at high levels of external visual white noise - a signature of improved external noise filtering. There were no significant effects of tRNS on task performance after the stimulation period. |
| **2.2.2 Auditory perception** | | | | | | |
| Prete et al., 2017 | Investigated whether tES can modulate illusory perception in the auditory domain. Hypothesized that the hyperactivity of the temporal cortex induced by tES could interfere with auditory processing, making the emergence of illusory percepts more difficult. | Exp.1 tDCS 60 healthy volunteers; c-tDCS: 30, 15F, 15M; 21.67±0.65; a-tDCS: 30, 15F, 15M; 20.33±0.19; 6 left-handed  Exp.2 tRNS 45 healthy volunteers; 28F, 17M; 22.67±0.42; all right-handed; no auditory impairments and no different hearing thresholds (±5 dBA) between left and right ears | tDCS:  5x7cm electrode over AC, between C3/4 and T3/4 sites (specifically C5 and C6 sites)  5x7cm electrode over contralateral shoulder  tRNS:  5x9.5cm electrode and  5x5cm electrode between C3/T3 and C4/T4 (specifically centered on C5 and C6 sites), AC | 2mA a-tDCS, c-tDCS for 20 min with 60s fade in/out  3 sessions: left AC, right AC, sham  1.5mA hf-tRNS (100-640Hz) with 0mA offset, for 20 min with 15s fade-in/out | 15s tDCS  15s tRNS | Hf-tRNS can modulate auditory perception more efficiently than tDCS.  Hf-tRNS applied bilaterally on the temporal cortex reduced the number of times the sequence of sounds was perceived as the Deutsch’s illusion with respect to the sham control condition.  Neither anodal nor cathodal tDCS applied over the left/right temporal cortex modulated the perception of the illusion. |
| Rufener et al., 2017 | Investigated AC tRNS-induced modulations in the participants’ temporal and spectral auditory resolution ability.  Measured gap detection task performance, pitch discrimination task performance and continuous EEG. | 20 healthy volunteers; 10F, 10M; age range: 20-35; all right-handed | 5x7cm electrodes horizontally over T7, and over T8 | 1.5mA hf-tRNS (100-640Hz) with 0mA offset, for 20 min (threshold evaluation), 15 min break, and 20 min (EEG + task) with 10s fade-in/out | 30s tRNS | Auditory tRNS increased the detection rate for near-threshold stimuli in the temporal domain only, while no such effect was present for the discrimination of spectral features.  Reduced peak latencies of the P50 and N1 component of the auditory event-related potentials indicating an impact on early sensory processing  The facilitating effect of tRNS was limited to the processing of near-threshold stimuli while stimuli clearly below and above the individual perception threshold were not affected by tRNS. |
| Prete et al., 2018 | Investigated the impact of hf-tRNS on auditory speech perception using the dichotic listening task.  Measured the right ear advantage effect, which in dichotic listening positively correlates with speech sound processing. | Exp.1 Bilateral  46 healthy volunteers; 31F, 15M; 22.79±0.41; all right-handed  Exp.2 Unilateral  24 healthy volunteers; 12F, 12M; 24.42±0.46; all right-handed; no auditory impairments and no different hearing thresholds (±5 dBA) between left and right ears | Exp.1 Bilateral montage: 5x9.5cm electrode and  5x5cm electrode over T3 and T4, AC  Exp.2 Unilateral montage: 5x5cm electrode over T3 or T4, AC 5x9.5cm electrode over contralateral shoulder | 1.5mA hf-tRNS (100-640Hz) with 0mA offset, for 20min with 15s fade-in/out  Exp.1 2 sessions separated at least 2h  Exp.2 3 sessions on 3 different days | 15s tRNS | Higher effectiveness of bilateral than unilateral hf-tRNS in modulating basic speech processing mechanisms.  Significant enhancement of the right ear advantage was found during bilateral (but not unilateral) hf-tRNS with respect to sham. |
| Rufener et al., 2018 | Investigated the involvement of the locus coeruleus-norepinergic system and a large-scale fronto-parietal cortical network in the regulation of auditory selective attention by applying tVNS and tRNS over the frontal cortex using auditory oddball paradigm and simultaneous EEG | 20 healthy volunteers; 10F, 10M; 24.85±2.62; age range: 21-30; normal hearing acuity | tRNS: 5x5cm electrodes over left DLPFC (F3), and over right shoulder | 1.5mA hf-tRNS (100-640Hz) with 0mA offset, for 30min (twice: first not part of this study, second oddball task), 10 min break, with 10s fade-in/out, started 5 min before the task  tVNS  0.5mA stimulation via the concha cymbae of the left ear; 25 Hz, pulse width 250 μs; with alternating on/off phases of 30 s; 90 min prior to the oddball task and lasted until the end, 100.5 min total.  3 sessions with 3 days break | 10s tRNS  10s tVNS | tRNS over the frontal cortex specifically modulates processes involved in stimulus evaluation and in the subjects’ behavioural response.  Compared to sham, tVNS increased the P3 amplitude, while tRNS reduced the response time to target stimuli. Moreover, both techniques reduced the P3 latency. |
| Rufener et al., 2020 | Investigated detection rate for near-threshold acoustic stimuli, with 3AFC task performance, during application of different levels of noise, either acoustically or electrically via tRNS. | Study 1 (acoustic noise): 29 healthy volunteers; 18F, 11M; 23.7±3.6  Study 2 (tRNS): 26 healthy volunteers; 16F, 10M; 24.5±3.9 | 5x7cm electrodes horizontally over T7, and over T8 over the left and right auditory cortex | 0.3, 0.6, 0.9, 1.2 and 1.5mA (amplitude) hf-tRNS (100-640Hz) delivered during task performance (6 blocks of different intensities; started 250 ms prior to the onset of the first observation interval and ended 250 ms after the offset of the last observation interval) | no tRNS or acoustic noise | Participants do not benefit from noise, irrespective of its modality (acoustic or tRNS). The results question the existence of SR in the human auditory system. |
| **2.2.3 Somatosensory perception** | | | | | | |
| Ambrus et al., 2010 | Investigated the cutaneous perception thresholds of tDCS and tRNS for current intensities ranging from 0.2 to 2mA | 30 healthy volunteers; 15F, 15M; 25.9±3.6  3 groups: 10 naïve to tES methods, 4F, 6M, 24.3±3.1; 10 subjects with previous experience with tES, 6F, 4M, 26.2±3.9 and 10 investigators, who use tES in their research, 5F, 5M, 27.4±3.4 | 3x3.5cm electrode over left supraorbital area, and over contralaterally C3, M1 | 0.2 – 2mA (with 0.1mA increments) tRNS (0.1-640Hz), a-tDCS, c-tDCS for 15 s with 8s fade-in/out per trial  3 sessions with 24h break  19 trials verum stimulation, + 7 non-stimulation trials | only fade-in/out of certain tES | tRNS as a possible alternative with a better blinding control. Higher cutaneous perception threshold for tRNS (1.2mA) than tDCS (0.4mA). Investigators better than naïve subjects in non-stimulation discrimination. |
| Ambrus et al., 2011 | Investigated the cutaneous perception differences for tDCS and tRNS between rectangle-shaped, and circle-shaped electrodes with the same surface area, and thus, same nominal current distribution. | 12 healthy volunteers; 6F, 6M; age range: 20-27 | 6.7cm diameter circle-shaped, round sponge wrappers, and the standard 5x7cm electrode wrappers for comparison. Both with an area of 35cm^2^; over left supraorbital area and contralaterally C3, M1 | 0.2 – 2mA (with 0.1mA increments) tRNS (0.1-640Hz), a-tDCS, c-tDCS for 15 s with 8s fade-in/out per trial  6 sessions with 24h break  19 trials verum stimulation, + 7 non-stimulation trials | only fade-in/out of certain tES | No difference between the round and the rectangular electrode configurations regarding their blinding potentials.  No substantial differences between detection thresholds, detection rates, false positive rates or consistent alterations in the sites of perceived stimulation. |
| **2.2.4 Motor function** | | | | | | |
| Jooss et al., 2019 | Investigated the task dependency of tRNS-induced neuromodulation in the motor system using a finger-tapping task versus a go/no-go task.  Measured single-pulse TMS-elicited MEPs before and after tRNS, finger-tapping and go/no-go performance assessed during and after tRNS | 30 healthy volunteers; 4 F, 12 M; 21±0.35; all right-handed | 12.5cm^2^ circular electrode over left M1, 30cm^2^ rectangular electrode over the contralateral frontopolar cortex | 1.51mA peak-to-peak (0.8 mA effective current intensity) of hf-tRNS (100-640Hz), with 0mA offset for 10 min  tRNS vs sham separated by at least 7 days | 15s fade-in/out tRNS | tRNS-induced neuromodulatory effects are task-dependent and the resulting enhancements are specific to the underlying task-dependent brain state. tRNS enhances the endogenous task-dependent brain state of healthy subjects. In an ‘activating’ motor task, tRNS during finger-tapping significantly facilitated corticospinal excitability. There was no difference in finger-tapping task performance between tRNS and sham stimulation. In an ‘inhibitory’ motor task, tRNS during go/no-go left corticospinal excitability was unchanged while inhibitory control was enhanced as shown by slowed reaction times and enhanced task accuracy during and after stimulation. |
| **2.2.5 Clinical: visual perception** | | | | | | |
| O’Hare et al., 2021 | Investigated whether tRNS modulates levels of internal noise in the brain of migraine patients and healthy controls.  Measured global motion direction discrimination task performance and visually based equivalent noise task performance. | 15 migraine patients, 11F, 4M; 30.93±10.85  16 healthy volunteers, 12F, 4M; 31.7±11.35; | 4x4cm electrodes over left and right hMT^+^ (3cm from the inion and 5cm to the right and left from this point) | 1.5mA hf-tRNS (100-600Hz) with 0mA offset for 20 min during task performance | 30s tRNS | Hf-tRNS can decrease internal noise levels in migraine.  The migraine group demonstrated increased baseline internal noise levels compared to the control group. Internal noise levels, and sampling, were reduced using hf-tRNS but not sham stimulation. There were no differences in terms of coherence thresholds, slopes, and lapse rate for global motion discrimination between the two groups. |
| **2.2.6 Clinical: motor function** | | | | | | |
| Hayward et al., 2017 | Investigated the feasibility of tRNS, timed to coincide with the generation of voluntary motor commands, during reaching training.  Measured the effects with adverse events, training outcomes, clinical outcomes, corticospinal tract structural integrity, and reflections on training through in-depth interviews from each individual case. | 4 stroke survivors with chronic (6-months to 5-years) and severe arm paresis; age range 49-73  tRNS: 2  sham: 2 | Electrodes over the ipsilesional M1 C3/C4 and over contralateral supraorbital region | 2mA tRNS with 0mA offset triggered to coincide with a voluntary movement attempt, for 5 s with 0s fade-in/out.  At this point, peripheral nerve stimulation enabled full range reaching.  12 sessions of reaching training (45min each) over 4-weeks | no tRNS | There were no adverse events. All training sessions were completed, repetitive practice performed and clinically relevant improvements across motor outcomes demonstrated. The amount of improvement varied across individuals and appeared to be independent of group allocation and corticospinal tract integrity. |
| **3. Potential cellular mechanisms of RNS** | | | | | | |
| Onorato et al., 2016 | Investigated how externally applied RNS influences action potential firing in mouse primary sensory neurons of dorsal root ganglia, modelling a basic process in sensory perception. Measured the effects of sub-threshold depolarizing current steps with superimposed random fluctuations. | 20 to 30 cultured neurons of dorsal root ganglia isolated from the full length of the spine, derived from four months old FVB and C57J mice of both sexes | Patch pipettes made of borosilicate glass for patch-clamp whole-cell recordings | RNS (variance scaled together with step) either before and during, or only during the 5 current depolarizing steps (0.5nA - 3.5nA) applied in alternation, for 30 or 40ms, delivered at 1 Hz | no RNS | External RNS enhances, via SR, the recruitment of transient voltage-gated Na^+^ channels, responsible for action potential firing in response to rapid stepwise depolarizing currents.  Stimuli of depolarizing step combined with RNS triggered significantly more action potentials than steps alone. The normalized power norm had a clear peak at intermediate noise levels, demonstrating that the phenomenon is driven by SR. |
| Remedios et al., 2019 | Investigated the physiological mechanism underlying electrical RNS. Measured the effects of short-term electrical noise applied to the voltage-clamp ramps on the kinetics of the Na^+^ current. Assessed correlation between the peak amplitude of the Na^+^ current and its latency for different levels of RNS. | 34 in-vitro, acutely-isolated brain pyramidal neurons from the somatosensory (N=16) and auditory (N=18) cerebral cortex of 7 Wistar rats (mean weight 100–150 g) | Glass microelectrode for whole-cell voltage clamp recordings | 0.045mV, 0.080mV, 0.142mV, 0.250mV, 0.445mV electrical RNS (0-5000Hz) for 250ms (short-term) delivered during Na^+^ currents eliciting six groups of 10 voltage-clamp-ramp protocol of 100ms, from −100 to +40 mV, with a holding potential of −80 mV | no RNS | There is an intermediate level of RNS that enhances the activation or inactivation processes occurring in the Na^+^ channels of the pyramidal neurons.  A Hodgkin–Huxley neuron model, involving the kinetics of activation and inactivation of the Na^+^ channels, explains differences in the impact of noise on three groups of pyramidal cells exhibiting a positive, negative or no correlation between peak amplitude of the Na^+^ current and its latency for different levels of RNS. |
| *no further methodological details were specified; Abbreviations: AC – auditory cortex; AFC – alternative forced choice; AMT – active motor threshold; ASSRs - auditory steady state responses; a-tDCS – anodal transcranial direct current stimulation; a-tPCS – anodal transcranial pulsed current stimulation; c-tDCS – cathodal transcranial direct current stimulation; CS - contrast sensitivity; DDM – drift-diffusion model; DLPFC - dorsolateral prefrontal cortex; EEG – Electroencephalography; ERSP - event-related spectral perturbation; fMRI – functional magnetic resonance imaging; hf-tRNS – high-frequency transcranial random noise stimulation; ICF – intracortical facilitation; IFC - interval forced choice; iTBS - intermittent theta- burst stimulation; lf-tRNS – low- frequency transcranial random noise stimulation; LICI - long-interval intracortical inhibition; M1 – primary motor cortex; MEP – motor evoked potential; MS – multiple sclerosis; PFC - prefrontal cortex; PSD - power spectral density; RMT – rest motor threshold; RNS – random noise stimulation; SEP-PPD - somatosensory evoked potential paired-pulse depression; SI_1mV_ – TMS intensity to evoke MEP of 1 mV peak-to-peak amplitude; SICI - short-interval intracortical inhibition; SR – Stochastic Resonance; tACS – transcranial alternating current stimulation; tDCS – transcranial direct current stimulation; TEP - transcranial magnetic stimulation evoked potentials; tRNS – transcranial random noise stimulation; TMS – transcranial magnetic stimulation; tVNS - transcutaneous vagal nerve stimulation; UCVA - uncorrected visual acuity; UCCS - uncorrected contrast sensitivity; V1 – primary visual cortex; VA – visual acuity; VEP - visual evoked potentials | | | | | | |
